# Acidic environments trigger intracellular H^+^-sensing FAK proteins to re-balance sarcolemmal acid–base transporters and auto-regulate cardiomyocyte pH

**DOI:** 10.1093/cvr/cvab364

**Published:** 2021-12-13

**Authors:** Abigail D Wilson, Mark A Richards, M Kate Curtis, Mala Gunadasa-Rohling, Stefania Monterisi, Aminah A Loonat, Jack J Miller, Vicky Ball, Andrew Lewis, Damian J Tyler, Anna Moshnikova, Oleg A Andreev, Yana K Reshetnyak, Carolyn Carr, Pawel Swietach

**Affiliations:** Department of Physiology, Anatomy & Genetics, University of Oxford, Sherrington Building, Parks Road, Oxford OX1 3PT, UK; Department of Physiology, Anatomy & Genetics, University of Oxford, Sherrington Building, Parks Road, Oxford OX1 3PT, UK; Department of Physiology, Anatomy & Genetics, University of Oxford, Sherrington Building, Parks Road, Oxford OX1 3PT, UK; Department of Physiology, Anatomy & Genetics, University of Oxford, Sherrington Building, Parks Road, Oxford OX1 3PT, UK; Department of Physiology, Anatomy & Genetics, University of Oxford, Sherrington Building, Parks Road, Oxford OX1 3PT, UK; Department of Physiology, Anatomy & Genetics, University of Oxford, Sherrington Building, Parks Road, Oxford OX1 3PT, UK; Department of Physiology, Anatomy & Genetics, University of Oxford, Sherrington Building, Parks Road, Oxford OX1 3PT, UK; Department of Physics, Clarendon Laboratory, University of Oxford, Parks Road, Oxford OX1 3PU, UK; Oxford Centre for Clinical Magnetic Resonance Research (OCMR), Radcliffe Department of Medicine, Level 0, John Radcliffe Hospital, Headington, Oxford OX3 9DU, UK; Department of Physiology, Anatomy & Genetics, University of Oxford, Sherrington Building, Parks Road, Oxford OX1 3PT, UK; Oxford Centre for Clinical Magnetic Resonance Research (OCMR), Radcliffe Department of Medicine, Level 0, John Radcliffe Hospital, Headington, Oxford OX3 9DU, UK; Department of Physiology, Anatomy & Genetics, University of Oxford, Sherrington Building, Parks Road, Oxford OX1 3PT, UK; Oxford Centre for Clinical Magnetic Resonance Research (OCMR), Radcliffe Department of Medicine, Level 0, John Radcliffe Hospital, Headington, Oxford OX3 9DU, UK; Physics Department, University of Rhode Island, 2 Lippitt Rd, Kingston, RI 02881, USA; Physics Department, University of Rhode Island, 2 Lippitt Rd, Kingston, RI 02881, USA; Physics Department, University of Rhode Island, 2 Lippitt Rd, Kingston, RI 02881, USA; Department of Physiology, Anatomy & Genetics, University of Oxford, Sherrington Building, Parks Road, Oxford OX1 3PT, UK; Department of Physiology, Anatomy & Genetics, University of Oxford, Sherrington Building, Parks Road, Oxford OX1 3PT, UK

**Keywords:** Acidity, Cardiomyocyte, Homeostasis, pH sensor, Ischaemia

## Abstract

**Aims:**

In cardiomyocytes, acute disturbances to intracellular pH (pHi) are promptly corrected by a system of finely tuned sarcolemmal acid–base transporters. However, these fluxes become thermodynamically re-balanced in acidic environments, which inadvertently causes their set-point pHi to fall outside the physiological range. It is unclear whether an adaptive mechanism exists to correct this thermodynamic challenge, and return pHi to normal.

**Methods and results:**

Following left ventricle cryo-damage, a diffuse pattern of low extracellular pH (pHe) was detected by acid-sensing pHLIP. Despite this, pHi measured in the beating heart (^13^C NMR) was normal. Myocytes had adapted to their acidic environment by reducing Cl^−^/${\text{HCO}}_{3}^{-}$ exchange (CBE)-dependent acid-loading and increasing Na^+^/H^+^ exchange (NHE1)-dependent acid-extrusion, as measured by fluorescence (cSNARF1). The outcome of this adaptation on pHi is revealed as a cytoplasmic alkalinization when cells are superfused at physiological pHe. Conversely, mice given oral bicarbonate (to improve systemic buffering) had reduced myocardial NHE1 expression, consistent with a needs-dependent expression of pHi-regulatory transporters. The response to sustained acidity could be replicated *in vitro* using neonatal ventricular myocytes incubated at low pHe for 48 h. The adaptive increase in NHE1 and decrease in CBE activities was linked to *Slc9a1* (NHE1) up-regulation and *Slc4a2* (AE2) down-regulation. This response was triggered by intracellular H^+^ ions because it persisted in the absence of CO_2_/${\text{HCO}}_{3}^{-}$ and became ablated when acidic incubation media had lower chloride, a solution manoeuvre that reduces the extent of pHi-decrease. Pharmacological inhibition of FAK-family non-receptor kinases, previously characterized as pH-sensors, ablated this pHi autoregulation. In support of a pHi-sensing role, FAK protein Pyk2 (auto)phosphorylation was reduced within minutes of exposure to acidity, ahead of adaptive changes to pHi control.

**Conclusions:**

Cardiomyocytes fine-tune the expression of pHi-regulators so that pHi is at least 7.0. This autoregulatory feedback mechanism defines physiological pHi and protects it during pHe vulnerabilities.

## Graphical Abstract

**Graphical Abstract:**
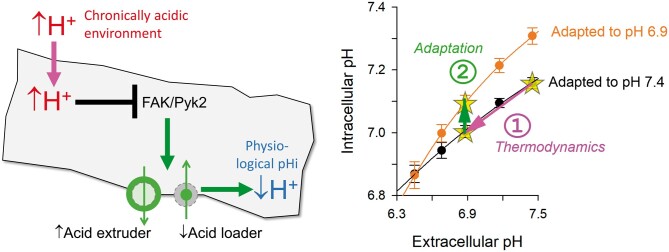

## 1. Introduction

As a result of an exquisite pH-sensitivity of protein function, many cardiac signalling pathways are only able to operate effectively over a narrow range of intracellular pH (pHi) centred around 7.1–7.2.^1,2^ Disturbances that push pHi outside this range have been documented to cause contractile depression,^3–7^ aberrant Ca^2+^ handling,^7,8^ and trigger electrical arrhythmias.^9^ To control pHi, cardiomyocytes are equipped with a system of H^+^-equivalent transporters,^10–14^ including Na^+^/H^+^ exchanger-1 (NHE1; *SLC9A1*),^15,16^ electrogenic Na^+^-HCO_3_^−^ cotransporter (*SLC4A4*),^17–20^ electroneutral Na^+^-HCO_3_^−^ cotransporter (NBCn1; *SLC4A7*),^18,19,21^ and Cl^−^/HCO_3_^−^ exchangers (CBEs; *SLC4A1-3*^18,22,23^ and *SLC26A6*^24–26^). This system regulates pHi towards a set-point, at which the H^+^-equivalent flux carried by acid-extruders (NHE1 and NBCs) balances the flux carried by acid-loaders (CBE). According to the canonical model, this regulatory system is sufficient to maintain a favourable pHi; for example, in response to an untoward cytoplasmic acid-load, intracellular H^+^ ions activate acid-extruders and inhibit acid-loaders, thereby restoring pHi within minutes. However, an inherent property of the proteins’ transport-cycle is that extracellular pH (pHe) also influences their activity.^24,27,28^ Consequently, extracellular acidosis inhibits acid-extruders and activates acid-loaders thermodynamically, thereby driving pHi to a lower level. Without a corrective mechanism, the internal acid–base milieu would become subservient to extracellular conditions, which is problematic because the interstitial fluid is susceptible to pH fluctuations, such as those arising from changes in perfusion during vascular development or myocardial disease.^29–31^ This regulatory flaw raises two questions: (i) is the cardiomyocyte able to offset thermodynamic pHe–pHi coupling and maintain internal homeostasis irrespective of the external milieu, i.e. is there a secondary level of pHi oversight that would be critical at times of reduced or aberrant vascular perfusion, and (ii) what instructs the pHi-regulatory apparatus to assemble in a way that produces a desired set-point, i.e. how does a cardiomyocyte determine what is normal pHi?

A plausible means of offsetting the undesirable coupling between pHe and pHi may involve an adaptive change to the expression of pHi-regulators, but how this takes place is unclear. Indeed, most of our understanding of how pHi-regulators are controlled relates to their post-translational status,^32–38^ i.e. a more acute and labile response that does not operate in the format of a pHi feedback circuit. A corrective mechanism would require an intracellular H^+^-sensor to instruct the appropriate expression of transporter-coding genes, but its identity in the heart is not established. Several candidates for such a sensor have been proposed, including histone (de)acetylase enzymes^39^ and the non-receptor kinases FAK1^40^ and FAK2 (also called Pyk2).^41,42^ Additionally, soluble adenylyl cyclases manifest an apparent pH-sensitivity because of their activation by HCO_3_^−^ ions.^43^ Aside from these intracellular enzymes, H^+^-sensing G-protein-coupled receptors^44^ (e.g. OGR1^45^) have been described in various cells; however, these probe extracellular conditions, which is not appropriate for the purpose of auto-regulating the internal milieu.

To address these questions, we studied the effect of sustained extracellular acidity on pHi regulation using an *in vivo* model of infarction, which produces a diffuse pattern of myocardial lactic acidosis,^46^ and then investigated the mechanism using a more tractable *in vitro* model of cultured myocytes adapted to acidic environments. We find that chronic exposure to extracellular acidity re-balances pHi control through changes in the expression of key pHi-regulator genes. This response is triggered by FAK1/Pyk2, an intracellular sensor of H^+^ ions, which operates a feedback circuit that titrates the appropriate levels of transporters required to attain a favourable pHi over a range of pHe. We thus describe a secondary level of pHi oversight that is mandated at times when pHe is unstable or abnormal.

## 2. Methods

### 2.1 Animal procedures

Animal experiments were approved by university ethical review boards and conform to the guidelines from Directive 2010/63/EU. For the cryo-infarct model, rats were anaesthetized by isoflurane (4% for induction and 2% for maintenance in O_2_) delivered by intubation. Pre-/post-operative analgesia was provided (buprenorphine and meloxicam). Animals were euthanized by an approved procedure listed under Schedule 1 of the Animals (Scientific Procedures) Acts 1986: isoflurane overdose (adult rats), cervical dislocation (adult mice and neonatal rats), confirmed by the removal of the heart (cessation of circulation).

### 2.2 Cryo-induced myocardial infarction model

Procedures were carried out under licence PPL30-3322 in compliance with the requirements of the UK Home Office (ASPA1986 Amendments Regulations 2012), which includes an explicit cost-benefit analysis and independent ethical review. Male, 6-week-old Sprague-Dawley rats were divided into cryo-injury or sham-surgery groups. Animals were anesthetized by isoflurane in oxygen (4% for induction and 2% for maintenance), intubated for ventilation, and maintained on a heated pad with monitoring of temperature, pulse oxygenation, and electrocardiogram (MouseMonitor S, Indus Instruments). Following a left thoracotomy and removal of the pericardium, the heart was stabilized by a loose stitch through the apex and myocardial infarction was induced by cryo-injury,^46,47^ via the placement of a 10 mm ø aluminium cylindrical probe cooled to 77 K onto the anterio-apical surface of the left ventricle for 15 s. The chest was closed in layers and the animal allowed to recover. In sham-operated rats, thoracotomy and cardiac exteriorization were performed, after which the chest was closed. All animals were provided with pre- and post-operative analgesia (buprenorphine and meloxicam) and lidocaine to prevent arrhythmia.

### 2.3 Oral bicarbonate supplementation

Procedures were carried out under licence PPL-P01A04016. Male, 7-week adult mice were given 400 mM bicarbonate water *ad lib* for 5 weeks. Control mice were housed separately and not given access to bicarbonate water.

### 2.4 ^13^C magnetic resonance

Hyperpolarized ^13^C magnetic resonance imaging (MRI) and magnetic resonance spectroscopy (MRS) were performed according to published methods, detailed in the Supplementary material online.

### 2.5 pHLIP imaging

pH-low insertion peptide (pHLIP) is a construct that undergoes a pH-dependent conformational change, favouring membrane bilayer insertion at low pH. It has been shown that as pHe falls below 6.5–7.0, pHLIP becomes anchored at the membrane.^48^ When conjugated with a fluorescent dye, the construct can identify areas of acidity in tissues by fluorescent microscopy. pHLIP peptide Var3 was synthesized and purified by CS Bio Co. pHLIP peptide and Cy5.5-maleimide (Lumiprobe) were dissolved in DMSO, as described in the Supplementary material online. Rats were tail-vein injected with a mixture of Var3 pHLIP fluorescently labelled with Cy5.5 [0.7 nmol/g in sterile phosphate buffered saline (PBS)] and Hoechst-33342 (10 mg/kg in sterile PBS) 5 h prior to tissue harvesting under licence PPL PF8462746. Animals were killed humanely by an approved Schedule 1 method and their hearts were excised, rinsed in PBS, blotted dry, and mounted in trays of OCT before flash freezing in powdered dry ice. Long-axis sections were cut on a cryostat at 25 µm thickness onto glass slides and stored at −80°C. Images were taken on a Leica DM6000 microscope with a motorized stage, using Volocity 6.4.0 (Quorum Technologies) for automatic tiling. pHLIP (excitation 683 nm/emission 703 nm) and Hoechst (excitation 350 nm/emission 461 nm) were imaged sequentially. pHLIP and Hoechst images were individually background-subtracted and then normalized to the mean Hoechst signal within myocardial regions.

### 2.6 Isolation of adult ventricular myocytes

Adult rat myocytes were isolated from hearts using enzymatic digestion using a previously published method,^49,50^ and kept in primary culture for up to 10 h. In some experiments, animals were injected with Hoechst-33342 (10 mg/kg in sterile PBS) 24 h prior to tissue harvesting under licence PPL PF8462746 to label myocytes *in vivo* according to perfusion status.

### 2.7 Neonatal ventricular myocyte culture

Myocyte isolation and culture was performed as described previously.^51^ Primary neonatal rat ventricular myocytes (NRVMs) were obtained from 1–2 day Sprague-Dawley rats euthanized by cervical dislocation. Cells were isolated by enzymatic digestion^52^ and a ‘pre-plating’ step was introduced to reduce fibroblasts in the myocyte-containing supernatant. Cells were plated onto fibronectin-coated tissue culture dishes or Ibidi slides, and cultured in medium (referred to as M2) made of 80% DMEM medium containing 24 mM NaHCO_3_ (D7777, Sigma/Merck) and 20% M199 medium with 26 mM NaHCO_3_ (M4530, Sigma/Merck) and incubated in a 5% CO_2_ atmosphere at 37°C. Medium was supplemented with 10% horse serum, 5% new born calf serum, and penicillin/streptomycin mixture. Next day, medium was replaced by serum-free M2 supplemented with insulin–transferrin–selenium (ITS) and penicillin/streptomycin for 24 h. NaHCO_3_ content was modified (2.2–24.4 mM) by iso-osmotic replacement with NaCl to achieve the desired pH.^53^

### 2.8 Measuring pHi with cSNARF

Myocytes were loaded with the acetoxymethyl ester of cSNARF1. When excited at a wavelength in the range 530–560 nm, cSNARF1 emits fluorescence that manifests a strongy pH-sensitive spectrum. By probing fluorescence at 580 and 640 nm, it is possible to record a ratio that is related to pH by the Grynkiewicz equation.^53^ The cSNARF1 ratio can be calibrated into units of pH by calibration experiments that use the H^+^/K^+^ ionophore nigericin, as described previously.^10^ This calibration will be unique to a given set-up.

### 2.9 High-throughput fluorescence imaging

pHi of cultured NRVMs was imaged in black walled, flat-bottom 96-well plates (Ibidi). Media was aspirated from wells and replaced with phenol-free media containing cSNARF1-AM (5 µg mL^−1^, Molecular Probes) and Hoechst-33342 (10 µg mL^−1^, Molecular Probes) for 15 min, and then replaced, twice, with dye-free medium. Images of fluorescence excited at 377 nm and collected at 447 nm (Hoechst), and of fluorescence excited at 531 nm and collected at 590 and 640 nm (cSNARF1), were acquired using Cytation 5 imaging plate reader (Biotek). All measurements were performed at 37°C. For media buffered with CO_2_/${\text{HCO}}_{3}^{-}$, measurements were performed in an atmosphere of 5% CO_2._^53^

### 2.10 pH imaging under superfusion

Adult myocytes were imaged in superfusion chambers coated with poly-L-lysine to improve cell adhesion. Neonatal myocytes were imaged as monolayers grown in Ibidi chambers. Live-cell imaging was performed on a Zeiss LSM 700 confocal system. Myocytes were loaded for 10 min with 20 µM 5-(and-6)-carboxySNARF-1-AM ester (ThermoFisher Scientific). After loading, superfusates were delivered at 37°C, and recordings were made once the steady-state was attained (∼10 min). cSNARF1 fluorescence was excited at 555 nm and measured at 580 and 640 nm. Hepes-buffered superfusates contained 135 mM NaCl, 4.5 mM KCl, 1 mM MgCl_2_, 1 mM CaCl_2_, 11 mM glucose, and 20 mM Hepes titrated to pH 7.4. CO_2_/${\text{HCO}}_{3}^{-}$ buffered superfusates were modified to contain 125 mM NaCl and NaHCO_3_ replaced Hepes, and the final solution was bubbled with 5% CO_2_ (balanced with air). Low-chloride or chloride-free solutions were prepared by replacing Cl^−^ salts with gluconate equivalents, and correcting for Ca^2+^ complexation by raising [CaCl_2_]. Ammonium- or acetate-containing solutions replaced NaCl with an equimolar amount of NH_4_Cl or NaAcetate, respectively.

### 2.11 Antibodies and western blotting

Lysates were prepared from cardiac tissue or NRVMs by fine homogenization or cell scraping in radioimmunoprecipitation assay buffer with a phosphatase–protease inhibitor cocktail (Roche) on ice. Lysates were centrifuged and protein concentration was assessed by BCA assay. Samples were resolved on a 10% reducing sodium dodecyl sulfate (SDS) polyacrylamide gel and blotted on a PVDF membrane. Membranes were blocked for 1 h at room temperature in 3% bovine serum albumin for phospho-antibodies or 5% low-fat milk for other antibodies in Tris-buffered saline and 0.1% Tween-20 (TBS-T). Primary antibodies were incubated overnight at 4°C. Antibodies used were: total-Pyk2 (CST 3292S, 1:1000); phospho-Pyk2 Y402 (Abcam 4800, 1:1000); phospho-Pyk2 Y579/580 (Invitrogen 44-636G, 1:1000); AE2 (Novus NBP159858, 1:500); and NHE1 (BD Biosciences 61175, 1:500). Membranes were washed with TBS-T and incubated with anti-rabbit/mouse HRP-conjugated secondary antibody (GE Healthcare Lifesciences). For loading controls, actin HRP-conjugated (Proteintech HRP60008, 1:20 000) and GAPDH HRP-conjugated (Proteintech HRP60004, 1:10 000) were used. Antibody–antigen complexes were visualized by Pierce™ enhanced chemiluminescent substrate with a Bio-Rad ChemiDoc™ Imaging System.

### 2.12 Statistics

Statistical testing of data involving myocytes was performed with hierarchical (nested) analysis.^54^ Briefly, measurements on adult myocytes are reported as number of cells/number of hearts that yielded cells. Data were nested based on the heart they were obtained from. Measurements on neonatal myocytes are reported as number of wells/number of isolations (each typically from 10 to 12 pups). Data were nested based on isolation batch. Statistical testing considered the degree of interclass clustering. RNAseq data were analysed by the DESeq2 package in R to identify significant hits with an adjusted *P*-value smaller than 0.05 and log fold-change of at least 0.5.

## 3. Results

### 3.1 Myocyte pHi undergoes a correction in acidic environments of infarcted hearts

**Figure 1:**
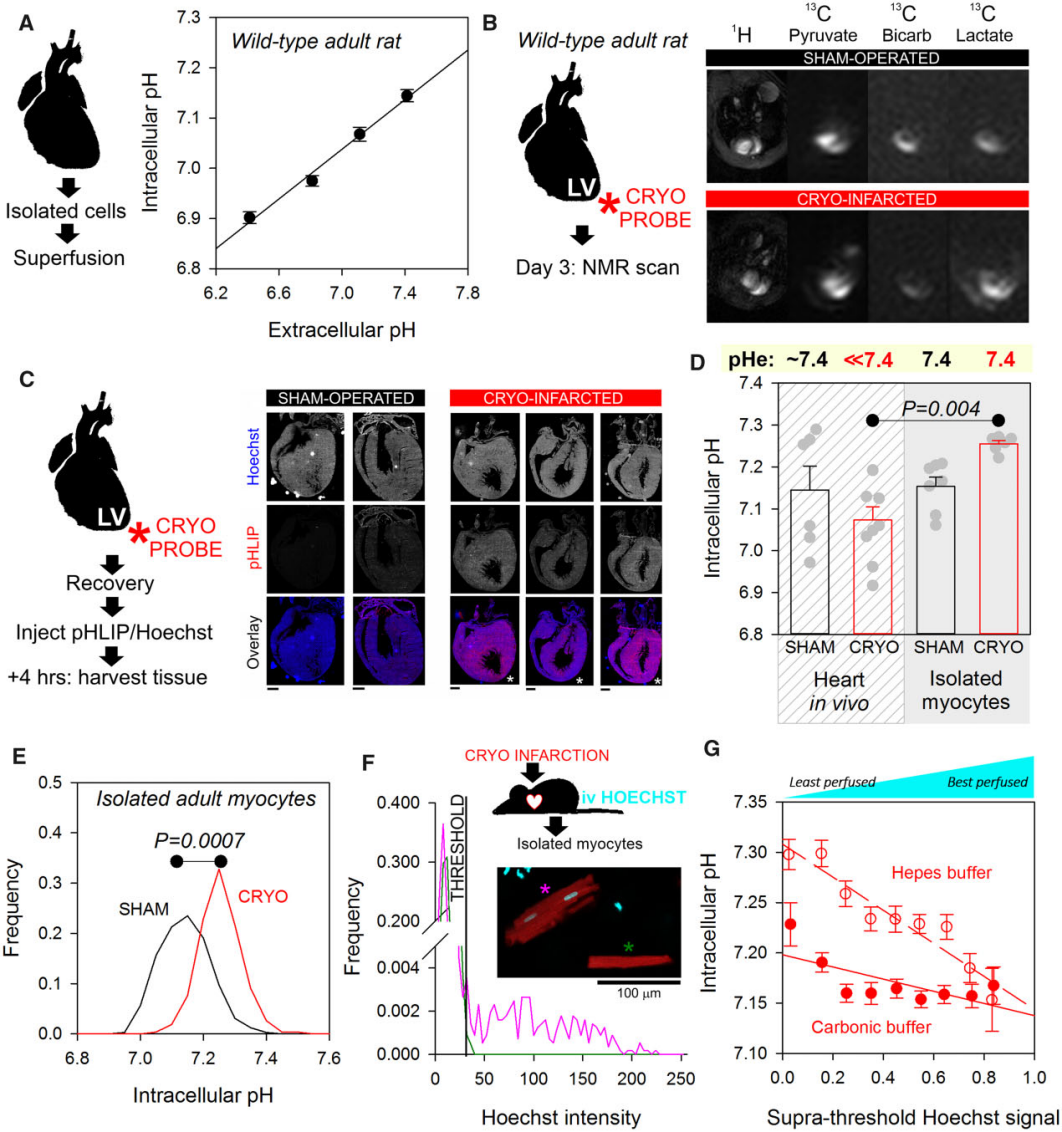
Hearts recovering from cryo-infarction sustain an acidic interstitium and adapt with a change in pHi control that maintains a favourable intracellular acid–base milieu. (*A*) Ventricular myocytes isolated from wild-type adult rats, imaged for pHi (cSNARF1) under superfusion with CO_2_/${\text{HCO}}_{3}^{-}$ buffered Tyrode. Superfusate pH changed by varying [${\text{HCO}}_{3}^{-}$]. Mean±SEM of 100–120 cells from five hearts. Best-fit line, pHi = 0.2469 pHe + 5.3093. (*B*) Exemplar image of metabolic response to cryo-infarction to left ventricle of adult rat. ^13^C metabolic imaging performed 3 days after surgery shows increase in lactate and decrease in bicarbonate over a diffuse area of the myocardium, compared to sham-operated hearts. (*C*) Second series of cryo-infarction experiments on adult rat hearts, allowing up to 5 weeks of recovery after injury. At 2 weeks post-surgery, animals were injected with a mixture of pHLIP and Hoechst and hearts harvested for sectioning and imaging. Cryo-infarcted hearts retained a greater degree of pHLIP fluorescence across the myocardium, indicating a diffuse pattern of extracellular acidosis. Exemplar images shown. Scale bar 1.6 mm. (*D*) At 5 weeks post-injury, pHi measured in beating hearts *in vivo* by ^13^C MRI, from the ratio of H^13^${\text{CO}}_{3}^{-}$ to ^13^CO_2_, compared to fluorescence (cSNARF1) measurements in superfused isolated myocytes. Mean±SEM from *N* = 6, 9, 7 (from total of 150 cells), 6 (from total of 134 cells) hearts. Significant difference by two-way ANOVA (*in vitro* v *in vivo* and cryo v sham). (*E*) Frequency histogram of pHi measured for myocytes from cryo-infarcted or sham-operated hearts 5 weeks post-injury. *N* = 150, 134 from 7 and 6 hearts, respectively. Hierarchical one-way ANOVA analysis, *P* = 0.0007. (*F*) cSNARF-loaded myocytes isolated from myocardium 5 weeks post-injury stained with Hoechst *in vivo* according to local perfusion status. Analysis of nuclear Hoechst-33342 staining, showing an exemplar cell with no nuclear staining (i.e. originating from under-perfused myocardial areas; green asterisk) and one with strong nuclear staining (i.e. derived from a well-perfused region; pink asterisk). (*G*) Correlation between nuclear Hoechst signal and resting pHi measured in the presence of CO_2_/${\text{HCO}}_{3}^{-}$ buffer (Pearson’s *P* = 0.0118) or absence of CO_2_/${\text{HCO}}_{3}^{-}$ buffer (*P* < 0.0001). *N* = 26–108 myocytes per bin, obtained from six hearts 5 weeks post-injury.

As a consequence of their transport-cycle, the ionic flux carried by sarcolemmal pHi-regulators responds acutely to changes in pHe. This results in a coupling between pHe and pHi at steady-state, which was measured in ventricular myocytes isolated from wild-type adult rats. Cells loaded with the pH-dye cSNARF1 were superfused with Tyrode solution over a range of pH, set by adjusting [HCO_3_^−^] at constant (5%) CO_2_. At steady-state, typically attained within 30 min, the pHe–pHi relationship was linear, with a gradient of 0.25, meaning that pHi will drop below 7.0 when pHe is <6.9 (*Figure 1A*). Thus, despite having a pHi-regulatory apparatus capable of generating H^+^-fluxes as large as several mM/min, steady-state pHi is subservient to pHe. Consequently, chronic exposure to acidic environments, such as under-perfused niches in the developing or diseased myocardium, would drive pHi to low levels, unless corrected by an adaptive process that overcomes the thermodynamic pHe–pHi coupling.

To seek *in vivo* evidence for a re-setting of pHi control in response to chronic acidosis, adult rat hearts were subject to cryo-infarction to the apex of the left ventricle.^47^ The technique produces a diffuse pattern of lactic acidosis across the myocardium and beyond the infarct region. The biological process underpinning this effect has been described previously.^46^ Briefly, the source of lactate detected by MRI is primarily the release from infiltrating macrophages, and some production also taking place in the blood. Lactate is a highly mobile anion, which leads to a diffuse appearance of its magnetic resonance signal across the heart. To confirm this spatial pattern in the present cohort of animals, lactate and bicarbonate were measured by hyperpolarized ^13^C MRI 3 days after cryo-infarction. The injury resulted in a diffuse build-up of lactate and a depletion of bicarbonate across a large part of the myocardium (*Figure 1B*). This pattern of lactate and bicarbonate is expected to result in diffusely distributed extracellular acidosis, which could trigger a pH-driven adaptive responses over a larger part of the myocardium. To test whether cryo-injury results in a diffuse pattern of chronic extracellular acidosis beyond the injury site, rats were administered a mixture of the acid-detecting peptide pHLIP and the nuclear stain Hoechst, delivered by tail-vein injection at 2 weeks post-surgery. After allowing 5 h for systemic distribution, hearts were harvested for sectioning and imaging. Fluorescence from Cy5.5-conjugated pHLIP was normalized to the tissue-averaged Hoechst signal and presented in *Figure 1C*, and quantified in Supplementary material online, *Figure S1*. Compared to sham-operated animals, there was a diffuse distribution of pHLIP fluorescence, indicating that large areas of the myocardium were acidotic at 2 weeks after surgery. Any form of adaptation to this chronic extracellular acidity would be expected to take place over a large area of the myocardium. Adaptive responses to chronic acidosis were measured at a later time point, to ensure sufficient time for their implementation. Based on prior studies reporting indices such as ejection fraction, the remodelling process evoked by cryo-injury is significantly resolvable by 5 weeks following surgery.^47,55^ For this reason, the 5 week time point was selected herein to seek evidence for an adaptation of pHi control to a period of sustained acidosis.

Myocardial pHi was measured *in vivo* by hyperpolarized [^13^C]-pyruvate MRI from the ratio of ^13^CO_2_ to H^13^CO_3_^−^ peaks in rats 5 weeks after cryo-injury or sham-surgery. Despite the low pHe reported by pHLIP, *in vivo* pHi measured by hyperpolarized ^13^C MRS was not significantly different to that determined in sham-operated hearts (*Figure 1D*). To test if this convergence in pHi reflects an adaptive re-setting of pHi regulation in hearts recovering from cryo-injury, enzymically isolated myocytes were imaged fluorescently for pHi under superfusion with CO_2_/HCO_3_^−^ buffer at pHe 7.4. In the case of hearts recovering from cryo-infarction, resting pHi was significantly higher in isolated myocytes under superfusion, compared to equivalent cells in the beating heart (*Figure 1D*). In contrast, the pHi in sham-operated hearts was no different between *in vivo* and *ex vivo* measurements. Taken together, these data indicate that myocytes in cryo-infarcted hearts had adapted to their acidic environment by correcting pHi, which becomes evident as a pHi overshoot upon superfusion at physiological pHe (*Figure 1E*).

The most profound adaptation of pHi control to acidic environments *in vivo* is expected in areas that are least perfused with blood. Myocytes derived from such under-perfused niches could be identified from nuclear Hoechst staining, after injecting the dye for systemic distribution prior to cell harvesting. Thus, 5 weeks following cryo-injury, rats were injected intravenously with a bolus of Hoechst, followed by enzymic isolation of cells 24 h later. Isolated myocytes emitting the strongest Hoechst signal would be derived from well-perfused areas. To quantify nuclear Hoechst signal, fluorescence collected within the cell outline was analysed for bimodality to determine a threshold that separates the low background in cytoplasm from the nuclear signal, if stained (*Figure 1F*). Signal summated above the threshold was normalized to total signal across the cell; this provided an index that quantifies the degree of nuclear staining, ranging from zero in myocytes derived from the least perfused regions of myocardium, to a high signal in cells from the best perfused regions (*Figure 1G*). There was a significant correlation between perfusion (as determined by Hoechst signal) and pHi, measured in the presence or absence of CO_2_/HCO_3_^−^ buffer. In other words, cells from the least perfused niches had undergone the most profound remodelling of pHi control. Thus, an adaptive process had taken place in myocytes *in situ* in response to inadequate perfusion. The outcome is a re-establishment of pHi homeostasis by overcoming the thermodynamic challenge arising from pHe–pHi coupling.

### 3.2 Adaptation to acidity involves a re-balancing of myocyte pHi control

**Figure 2:**
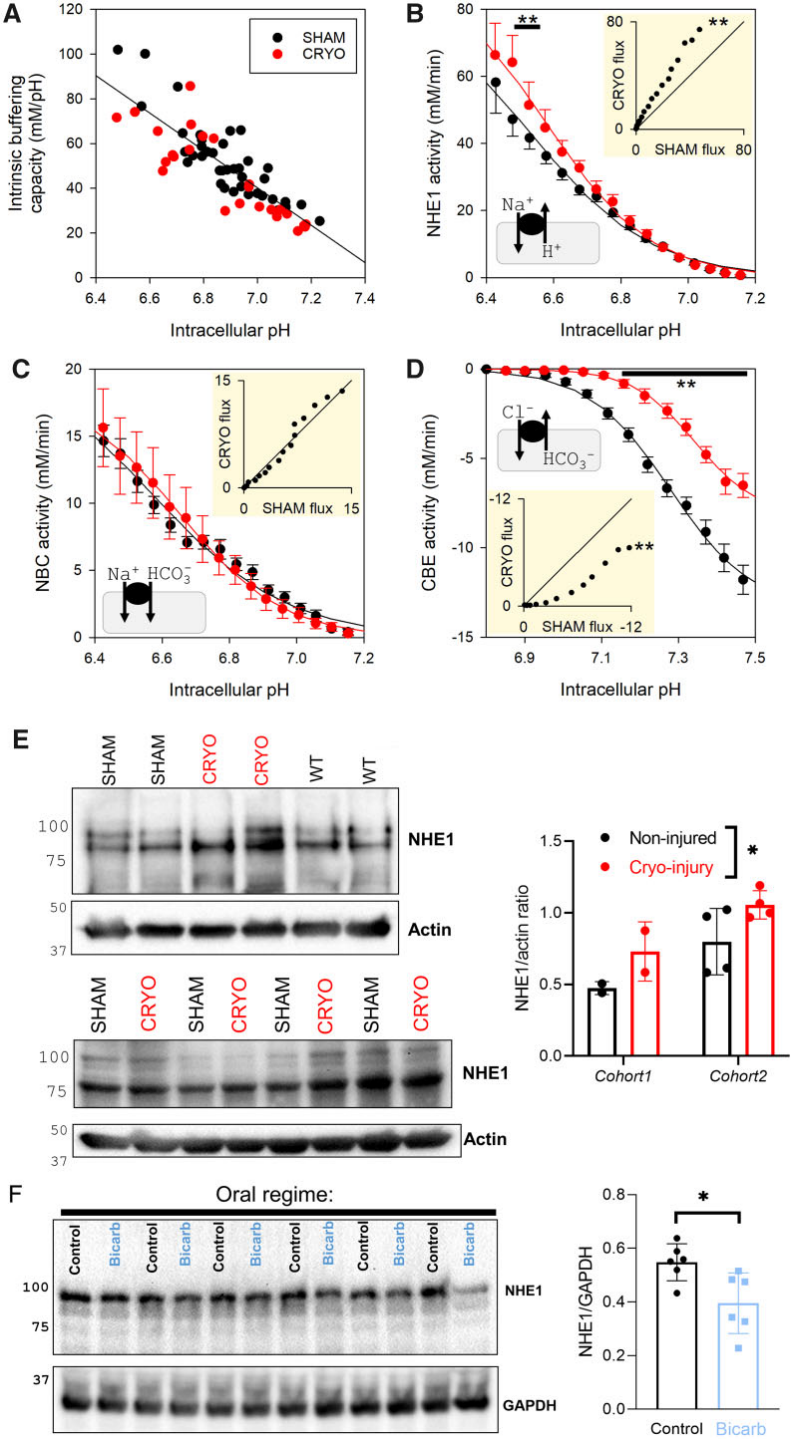
*In vivo* acid-adapted cardiac myocytes manifest altered pHi regulation. (*A*) Intrinsic buffering capacity measured by stepwise ammonium removal protocol in myocytes superfused with Hepes-buffered solutions. Data from 44 myocytes from 6 sham-operated hearts and 25 myocytes from 6 cryo-infarcted hearts. (*B*) NHE1 activity measured by ammonium prepulse using Hepes-buffered solutions. Data from 54 myocytes from six sham-operated hearts and 25 myocytes from six cryo-infarcted hearts. (*C*) NBC activity measured by ammonium prepulse using CO_2_/${\text{HCO}}_{3}^{-}$ buffered solutions. Data from 74 myocytes from six sham-operated hearts and 30 myocytes from six cryo-infarcted hearts. (*D*) CBE activity measured by acetate prepulse using CO_2_/${\text{HCO}}_{3}^{-}$ buffered solutions, with a 2 min interval in chloride-free solution to stabilize CO_2_/${\text{HCO}}_{3}^{-}$ buffering prior to CBE activation. Data from 91 myocytes from six sham-operated hearts and 36 myocytes from six cryo-infarcted hearts. Significance testing by hierarchical two-way ANOVA shows significant effect of infarction (vs. sham) on NHE1 (*P* < 0.01) and CBE (*P* < 0.01) activities, in addition to a significant effect of pH (*P* < 0.001). (*E*) Western blots for NHE1, showing expression in lysates prepared from sham-operated, cryo-infarcted, and non-operated, wild-type hearts in Cohort 1 and sham-operated and cryo-infarcted hearts in Cohort 2. Densitometric analysis of NHE1 expression, showing significant increase in NHE1 levels in cryo-injured hearts (*n* = 2 in Cohort 1; 4 in Cohort 2) relative to sham-operated hearts (*n* = 2; 4; *P* = 0.0401; determined by nested ANOVA) (*F*) Western blot for NHE1, showing expression in lysates from mice given a course of oral bicarbonate vs. controls. Densitometric analysis, showing significant difference (*P* < 0.05; determined by *t*-test).

The re-setting of pHi in the infarcted heart must involve a re-balancing of fluxes carried by sarcolemmal acid-base transporters. To characterize this, H^+^-equivalent transport was measured in myocytes isolated from cryo-infarcted or sham-operated hearts at 5 weeks after surgery to allow adaptive processes to take place. In order to calculate flux, intrinsic pH buffering was measured using a stepwise ammonium removal protocol^10^ (*Figure 2A*). Buffering was no different between sham and cryo-infarcted heart, and therefore a pooled buffering line was used for flux analyses. NHE1 activity was measured by the ammonium prepulse method in the absence of CO_2_/HCO_3_^−^ (*Figure 2B*) and NBC activity was determined in the presence of CO_2_/HCO_3_^−^ and 30 µM dimethyl amiloride (*Figure 2C*). CBE activity was determined by acetate prepulse, with an intermediate step in chloride-free solution to allow CO_2_/HCO_3_^−^ buffer equilibration prior to acid-loading^11^ (*Figure 2D*). Hierarchical statistical analyses showed that myocytes from cryo-infarcted hearts produced significantly smaller acid-loading by CBE but higher acid-extrusion by NHE1, whereas NBC activity was unchanged. This increase in the ratio of NHE1-to-CBE activity is a means of offsetting set-point pHi.

Lysates prepared from cryo-damaged hearts had higher NHE1 immunoreactivity relative to sham controls and un-operated age-matched hearts, indicating that the increase in NHE1-carried flux involves, at least in part, a change in expression (*Figure 2E*). This effect may relate to a myriad of changes associated with infarction, so to test if NHE1 expression was generally responsive to pHe, a series of experiments were performed on animals with raised systemic buffering, which produces a more stable pHe environment for myocytes in the heart. To attain this, mice were given bicarbonate in drinking water *ad lib* for 5 weeks.^56^ At the end of the protocol, cardiac lysates were prepared for immunoblotting. Higher systemic buffering reduced NHE1 immunoreactivity relative to control mice, consistent with a more stable pHe (*Figure 2F*). Taken together with observations made on hearts recovering from infarction, the findings indicate that the pHi-regulatory apparatus responds to sustained changes in ambient pHe in both directions.

### 3.3 Adaptation of pHi control to acidic environments involves changes in *Slc4a2*, *Slc9a1*, and *Slc4a7* expression

**Figure 3:**
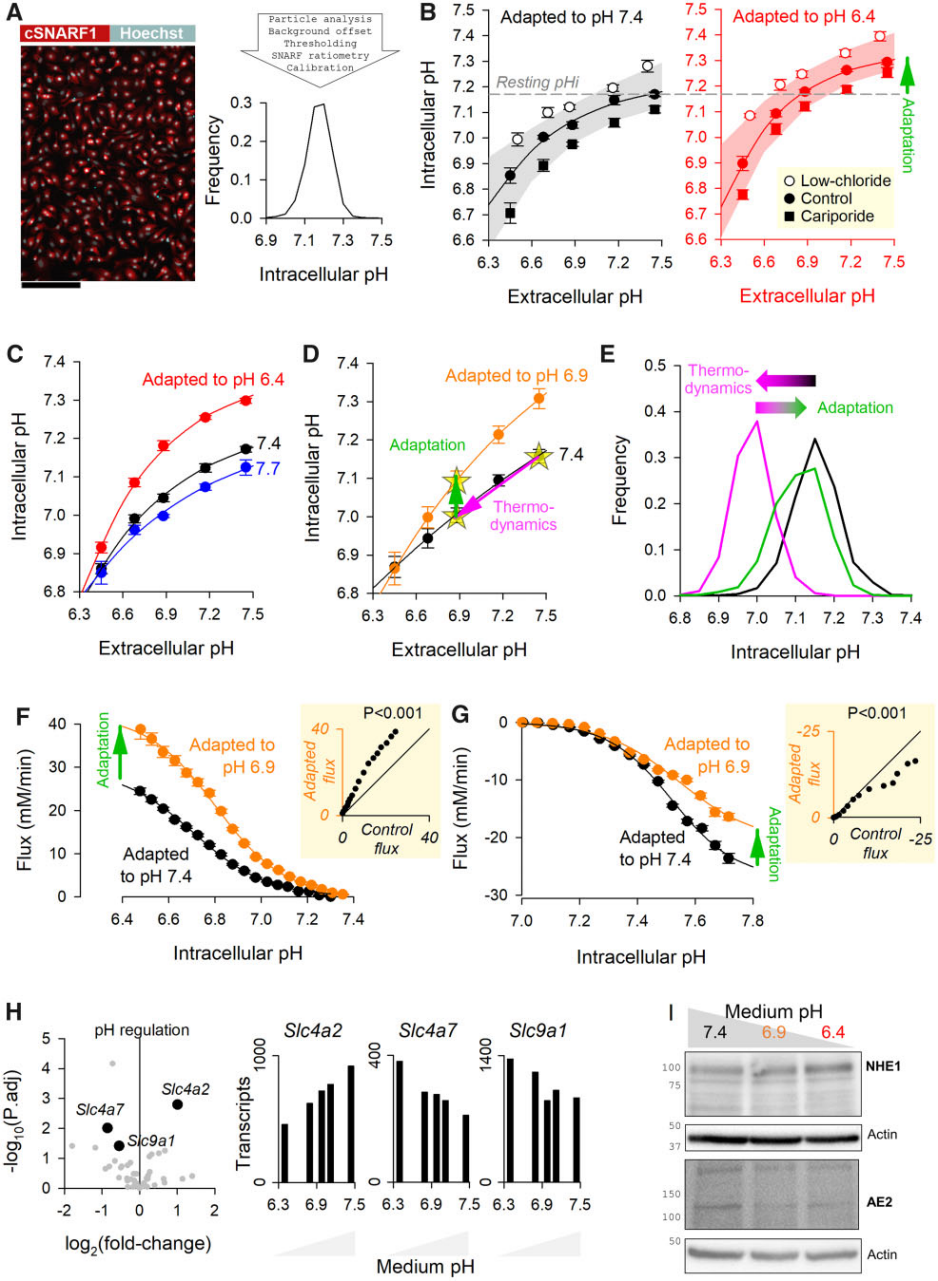
*In vitro* acid-adaptation of NRVMs remodels pHi regulation through a shift in gene expression favouring acid-extruders. (*A*) High-throughput imaging of pHi in cultured NRVMs. Scale bar is 200 µm. Image analysis pipeline produces a statistical distribution of pHi. (*B*) Steady-state pHi measured over a range of pHe varied acutely by changing medium [${\text{HCO}}_{3}^{-}$]. Relationship was mapped following 4 h equilibration in media containing 30 µM cariporide or in low-chloride media. Experiments performed on control myocytes (incubated at pHe = 7.4 for 48 h) or acid-adapted myocytes (incubated at pHe = 6.4 for 48 h). Significant alkaline shift for matching conditions by two-way ANOVA (*P* < 0.01). Mean±SEM of the average value from four biological repeats (isolations), each performed with four technical replicates. In all panels, experiments were paired from the same isolation batch. (*C*) pHe–pHi relationship for myocytes adapted to pHe 6.4, 7.4, or 7.7 for 48 h. Mean±SEM of the average value from three biological repeats (isolations), each performed with four technical replicates. (*D*) pHe–pHi relationship for myocytes adapted to pHe 6.9 or 7.4 for 48 h. Mean±SEM of the average value from eight biological repeats (isolations), each performed with four technical replicates. Arrows illustrate the iterative process that myocytes experience in low pHe: initial thermodynamically driven pHi acidification, followed by a gradual re-active adaptation. (*E*) Histograms of pHi from an exemplar experiment from (*D*). (*F*) Acid-extrusion flux measured by ammonium prepulse in CO_2_/${\text{HCO}}_{3}^{-}$ buffered superfusates on control and acid-adapted myocytes. Mean±SEM of 496/602 cells from eight isolations. Significant effect of acid-adaptation by hierarchical two-way ANOVA. (*G*) Acid-loading flux measured by acetate prepulse in CO_2_/${\text{HCO}}_{3}^{-}$ buffered superfusates on control and acid-adapted myocytes (with a 2 min resting period in chloride-free solutions before activation of transport). Mean±SEM of 802/1013 cells from eight isolations. Significant effect of acid-adaptation by hierarchical two-way ANOVA. (*H*) Analysis of RNAseq experiment on myocytes incubated for 48 h at five levels of pH between pH 6.4 and 7.4 (triplicates per condition). Genes belonging to ‘intracellular pH regulation’ gene ontology GO: 0051453. Analysis by DESeq2 shows significant correlation between medium pH and *Slc4a2* (positive), *Slc4a7* (negative), and *Slc9a1* (negative) transcripts. (*I*) Western blot showing up-regulation of NHE1 and down-regulation of AE2 in acid-adapted cells.

Cultured neonatal ventricular myocytes (NRVMs) have been established as a tractable model for studying various molecular mechanisms. To determine their suitability for investigating the mechanism of acid-adaptation, it was first necessary to demonstrate that the *in vivo* actions of chronically low pHe on pHi control could be replicated *in vitro*. NRVMs were incubated at pHe 6.4 (acid-stimulus) or 7.4 (control) for 48 h, and then dually loaded with cSNARF1 (to measure pHi) and Hoechst (to identify nuclei) for imaging. Fluorescence images were acquired on a high-throughput imaging platform, and an offline analysis pipeline generated the statistical distribution of pHi (*Figure 3A*). The pHi-regulatory apparatus of myocytes was interrogated in terms of its acute pHe-sensitivity (i.e. pHe–pHi coupling), determined after allowing ∼30 min for equilibration with media over a range of pHe (6.4–7.4), attained by varying [HCO_3_^−^] at constant (5%) CO_2_. Parallel experiments were performed in the presence of 30 µM cariporide added 4 h prior to imaging to block the contribution from NHE1, and in low-chloride media replaced 4 h before imaging to hinder acid-loading flux carried by CBE. To characterize the effect of long-term acidosis on pHi control, measurements were performed on myocytes that had been incubated for 48 h at pH 6.4 (acid-adapted) or 7.4 as its control (*Figure 3B*). In general, pHe–pHi curves shifted downwards with cariporide and upwards in low-chloride media, confirming that steady-state pHi is set by the balance between acid-extruding NHE1 and acid-loading CBE. After 48 h in acidic media, these pHe–pHi curves shifted in the alkaline direction, indicating that pHi control had adapted to the prior acidic incubation. Notably, the pHe–pHi relationship became more curved in acid-adapted myocytes, which ensures a more alkaline pHi over a wider range of pHe. For example, myocytes that had been incubated at pHe 7.4 were able to maintain pHi above 7.0 over acute pHe-disturbances down to pHe 6.7, whereas acid-adapted myocytes could do so over a wider pHe range, down to pHe 6.5. This adaptation confers a clear advantage, as it enables cardiac functions to operate in ther optimal pHi-range, even when the cellular environment becomes chronically acidity.

Additional pHe–pHi curves were measured for NRVMs adapted to a range of pHe (*Figure 3C and D*). In general, adaptation to acidic media tended to increase the steepness and the curvature of pHe–pHi curves. The events that result in these outcomes can be summarized in terms of an iterative process, shown in *Figure 3D*. Initially, exposure to an acidic environment thermodynamically drives pHi to a lower level; over time, cells respond to the sustained acidosis through an adaptive process that offsets, albeit partially, the original thermodynamic challenge. *Figure 3E* illustrates this process using frequency distributions of pHi measured under control conditions, in response to an acute displacement of pHe to 6.9, and following 48 h adaptation to pH 6.9.

The mechanism underpinning the adaptation to acidity was interrogated functionally in terms of acid-extrusion and acid-loading fluxes, measured by ammonium and acetate prepulse, respectively. To make comparisons at matching conditions, experiments used CO_2_/HCO_3_^−^ buffered superfusates at pH 7.4. Acid-adapted NRVMs presented with higher acid-extrusion (*Figure 3F*) but lower acid-loading fluxes, relative to time-matched controls incubated at pH 7.4 (*Figure 3G*). This re-balancing explains how the set-point pHi increases in acid-adapted myocytes. Since these measurements were performed in superfusates at pH 7.4 within 1 h of withdrawing the acid-stimulus, the effect of acid-adaptation must involve a sustained change in transporter activity, such as a change in the expression of genes coding for acid–base transporters. Analysis of RNAseq transcriptomics of NRVMs treated for 48 h in pHe ranging from 6.4 to 7.4 identified three pHe-responsive genes implicated in pHi control: *Slc4a2* (coding for AE2), *Slc9a1* (coding for NHE1), and *Slc4a7* (coding for NBCn1). As part of adaptation to low pHe, expression of acid-loading *Slc4a2* decreased whereas expression of acid-extruding *Slc9a1* and *Slc4a7* increased (*Figure 3H*). The response of *Slc9a1* and *Slc4a2* is consistent with data for NHE1 and CBE fluxes measured in the cryo-infarct model, and was confirmed at protein level in NRVMs by western blot, showing up-regulation of NHE1 protein and down-regulation of AE2 protein (*Figure 3I*; quantified in Supplementary material online, *Figure S2*).

### 3.4 Acid-adaptation of pHi control is instigated by FAK-family intracellular H^+^-sensors

**Figure 4:**
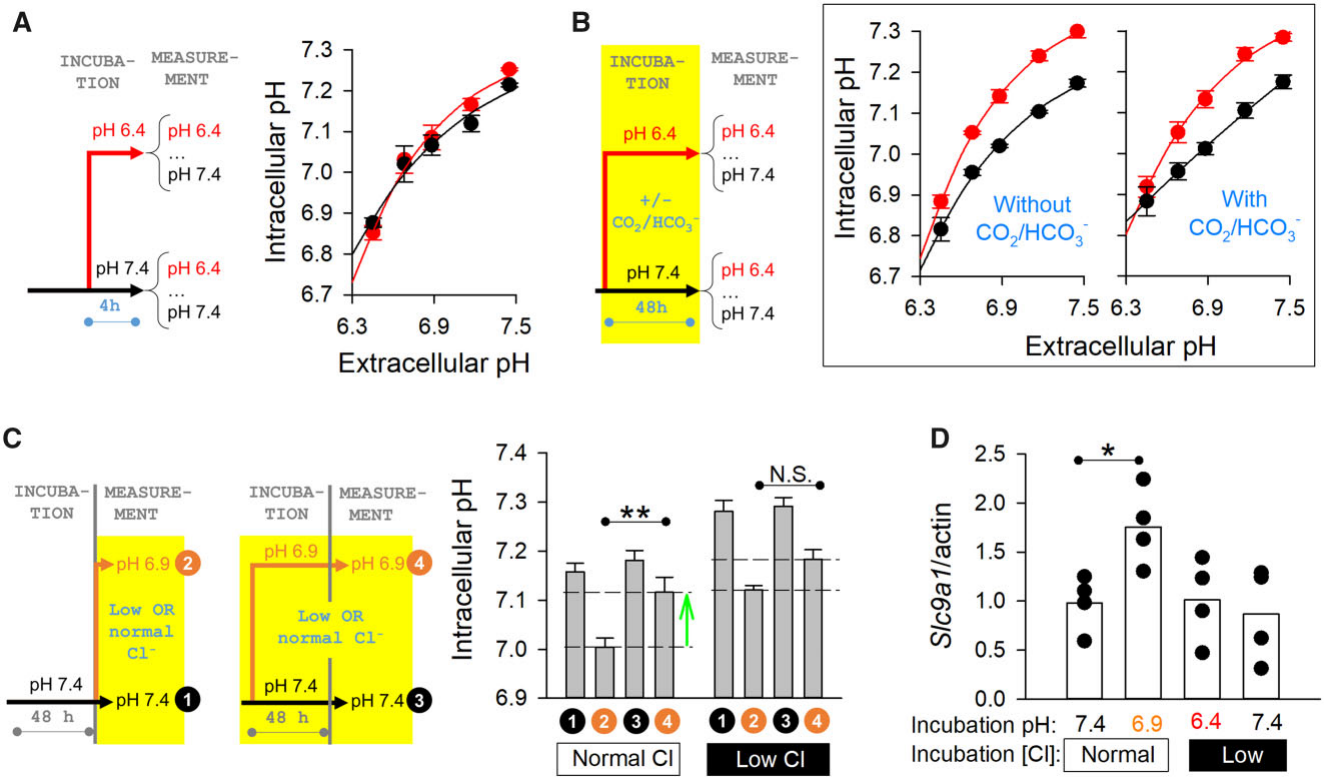
Acid-adaptation of pHi control is a slow-onset process triggered by an intracellular H^+^ sensor. (*A*) Steady-state pHi measured over a range of pHe varied acutely in CO_2_/${\text{HCO}}_{3}^{-}$ buffered media by changing [${\text{HCO}}_{3}^{-}$]. Experiments performed on myocytes exposed for 4 h to low pHe (6.4), and compared to time-matched controls at pH 7.4. No significant effect of 4 h acid-treatment. Mean±SEM of the average value from three biological repeats (isolations), each performed with four technical replicates. Experiments were paired from the same isolation batch. (*B*) pHe–pHi curves mapped in CO_2_/${\text{HCO}}_{3}^{-}$ buffered media after 48 h of acid-adaptation (or control pHe) in the presence or absence of CO_2_/${\text{HCO}}_{3}^{-}$. No significant effect of removing CO_2_/${\text{HCO}}_{3}^{-}$ during adaptation. Mean±SEM of the average value from three biological repeats (isolations), each performed with four technical replicates. Experiments were paired from the same isolation batch. (*C*) pHi measured in low- or normal-chloride media following a 48 h period of acid-adaptation or control in low- or normal-chloride media. All experiments performed in presence of CO_2_/${\text{HCO}}_{3}^{-}$. Mean±SEM of the average value from three biological repeats (isolations), each performed with four technical replicates. Experiments were paired from the same isolation batch. (*D*) qPCR measurements of *Slc9a1* following 48 h acid-adaptation or time-matched controls in lowor normal-chloride media. Mean±SEM of the average value from four biological repeats (isolations). Experiments were paired from the same isolation batch.

To investigate the time course of the acid-adaptation response, experiments were performed on NRVMs exposed to acidic media (pH 6.4) for a shorter, 4 h period. This protocol was not sufficient to fully develop the acid-adaptation response to 48 h acidity (*Figure 4A*), which is consistent with acid-adaptation being a slow-onset process, such as involving a change in gene expression. The mechanism of acid-adaptation was investigated further in myocytes subjected to various interventions during the 48 h incubation period. The underlying sensing mechanism may gauge pH directly from the level of H^+^ ions, or indirectly from HCO_3_^−^ ions. A precedent for the latter is CO_2_/HCO_3_^−^ sensitive soluble adenylyl cyclases residing intracellularly and receptor tyrosine phosphatase γ (RTPγ), which presents an exofacial HCO_3_^−^ sensor. To distinguish these alternative sensor ligands, acid-adaptation was performed in the absence of CO_2_/HCO_3_^−^, replacing this buffer with an equimolar mixture of Hepes and Mes (in 0% CO_2_). At the end of the incubation, the pHe–pHi relationships were mapped in the presence of CO_2_/HCO_3_^−^ buffer, and compared with controls that had been acid-adapted in CO_2_/HCO_3_^−^ throughout. In the nominal absence of CO_2_/HCO_3_^−^ during incubation, a 48 h period in acidity was still able to shift the pHe–pHi relationship upwards, indicating that the sensor is triggered by H^+^ ions, and not components of CO_2_/HCO_3_^−^ (*Figure 4B*).

The change in the expression of genes responsible for pHi-regulation may be triggered by an exofacial sensor, detecting low pHe, or an intracellular sensor that probes pHi directly. Extracellular facing receptors have been widely studied in various tissues, and include receptors for H^+^ ions, such as the G-protein-coupled receptor OGR1. Strategically, however, an *intracellular* sensor would be best placed to gauge the outcome of a cellular adaptation to pHe. To distinguish these alternative locations, NRVMs were incubated in low-chloride media, a manoeuvre that raises pHi at constant pHe as a result of loading cytoplasm with HCO_3_^−^ ions. By means of this solution change, it is possible to expose NRVMs to acidic conditions for 48 h, without evoking the full extent of the pHi decrease. The protocols for this experiment are shown in *Figure 4C*. Cells were adapted to pH 6.9 or 7.4 in either low- or normal-chloride media for 48 h, followed by measurements at matching conditions (in CO_2_/HCO_3_^−^ buffer). The controls for these experiments were cells that had been incubated in pH 7.4 in normal-chloride media, and then probed at 6.9 or 7.4 in either low- or normal-chloride media. In normal-chloride media, incubation at low pHe evoked the expected adaptive response (shown by green arrow). However, this effect was absent in parallel experiments performed under low-chloride conditions. Together, these findings implicate an intracellular H^+^ sensor, which becomes engaged when pHi falls, but not when cells are HCO_3_^−^-overloaded in low-chloride media (*Figure 4C*). To verify this observation at the level of gene expression, qPCR measurements of *Slc9a1* were performed in NRVMs cultured at 7.4 or at 6.9 in normal-chloride media or at 7.4, 6.9, or 6.4 in low-chloride formulations (*Figure 4D*). The gene coding for NHE1 (*Slc9a1*) was up-regulated only under conditions that allowed pHi to fall substantially, i.e. acidic media containing normal chloride, but not when pHi was raised in low-chloride formulations.

**Figure 5:**
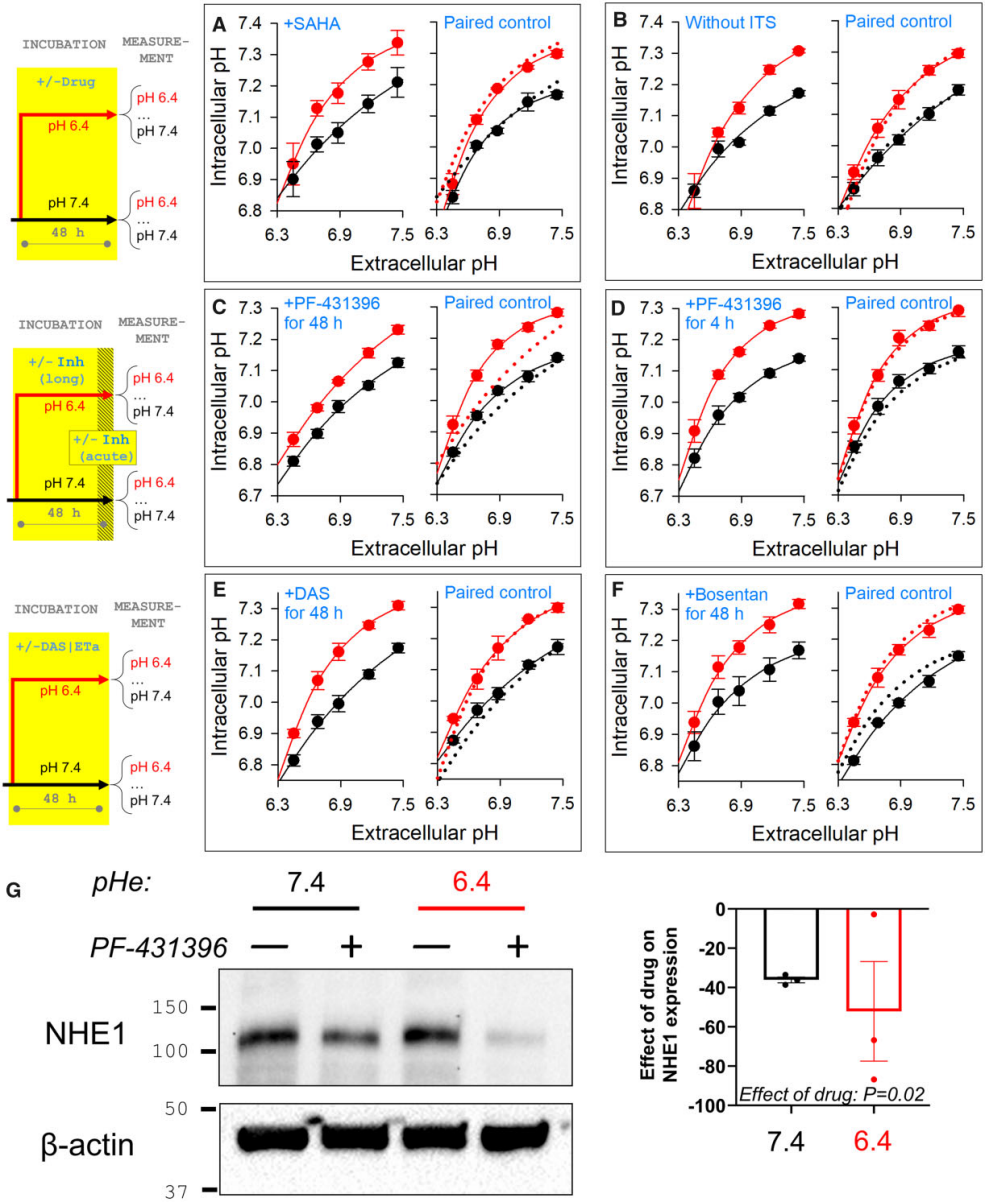
Acid-adaptation of pHi control is transduced by FAK-family H^+^ sensors. Steady-state pHi measured over a range of pHe varied acutely in CO_2_/${\text{HCO}}_{3}^{-}$ buffered media by changing [${\text{HCO}}_{3}^{-}$]. Experiments performed on acid-adapted myocytes (48 h in pH 6.4) or control myocytes (48 h in pH 7.4). Each biological repeat was performed with four technical repeats, and experiments were paired from the same isolation batch. (*A*) Effect of 10 µM SAHA. No significant effect. Mean±SEM of the average value from three biological repeats (isolations). (*B*) Effect of removing ITS. No significant effect. Mean±SEM of the average value from three biological repeats (isolations). (*C*) Effect of 10 µM PF-431396 (FAK1/Pyk2 inhibitor) included during 48 h acid-adaptation or control incubation. Significant effect of inhibitor (*P* < 0.001) and significant interaction with incubation pH (*P* < 0.01) on three-way ANOVA. Mean±SEM of the average value from three biological repeats (isolations). (*D*) When added for final 4 h period of acid-treatment and during imaging, PF-431396 had no significant effect. Mean±SEM of the average value from three biological repeats (isolations). (*E*) Effect of 100 nM Dasatinib during acid-adaptation (or time-matched controls). No significant effect. Mean±SEM of the average value from three biological repeats (isolations). (*F*) Effect of 10 µM Bosentan during acid-adaptation (or time-matched controls). No significant effect. Mean±SEM of the average value from three biological repeats (isolations). (*G*) Western blot showing down-regulation of NHE1 in cells with the addition of 10 µM PF-431396 (FAK1/Pyk2 inhibitor) in acid-adaptation or control incubation cells. Effect of drug tested by two-way ANOVA; significant effect of drug (mean of three blots).

There is a myriad of candidates for the intracellular H^+^ sensor, which may take the form of a discrete receptor, or be devolved among many proteins collectively manifesting pHi-sensitivity. A notable pH-dependent process that feeds into gene expression is histone acetylation. To test if adaptation to low pHe is dependent on a change in acetylation, a broad-spectrum histone deacetylase inhibitor, SAHA (10 µM), was applied during the 48 h incubation period at pH 6.4 or 7.4. Acetylases are generally inhibited at low pH, but this effect would be cancelled-out in the presence of SAHA. However, SAHA did not affect the acid-adaptation response (*Figure 5A*).

A plausible mechanism for regulating SLC-type genes may involve kinases. Among the available transcriptomics datasets describing the effect of twenty-six FDA-approved kinase inhibitors on gene expression in human cardiac cells,^57^ several drugs were found to affect the expression of at least one of the genes involved in acid-adaptation (*SLC9A1, SLC4A2* and *SLC4A7*) (Supplementary material online, *Table S1*). Thus, kinase-operated pathways are candidates for transducing a signal of sustained acidosis onto a change in pHi regulation. Culture media for NRVMs are, per standard protocol, supplemented with ITS, and the operation of its downstream signalling pathway may endow pH-sensitivity. However, removing ITS in the 48 h acid-incubation period had no effect on acid-adaptation outcomes (*Figure 5B*).

Kinases that have been ascribed a *bona fide* pH-sensing role include two members of the FAK-family, FAK1^40^ and FAK2 (Pyk2).^41,42^ These non-receptor tyrosine kinase^58–60^ have a histidine-rich FERM domain believed to mediate the effect of pHi on auto-phosphorylation. The FAK1/Pyk2 inhibitor PF-431396 (10 µM), when included for the duration of acid-treatment, ablated the acid-adaptation response of myocyte pHi control. In particular, the ensuing pHe–pHi relationship lacked the characteristic curvature normally attained with acid-adaptation (*Figure 5C*). This effect was not observed when PF-431396 was added for the final 4 h of acid-treatment and during imaging (*Figure 5D*), indicating that the inhibitor must target an early step in the acid-adaptation response in order to produce its effect. Src kinases are part of the FAK signalling pathway, and have been implicated as the enzymes responsible for phosphorylating FAK1 and Pyk2 following their auto-phosphorylation. However, the Abl/Src kinase inhibitor Dasatinib (100 nM) did not phenocopy the effect of PF-431396, suggesting that H^+^ ions act at the level of FAK auto-phosphorylation (*Figure 5E*).

Of the two FAK-family kinases, a series of renal studies^41,42^ implicated Pyk2 in a feedback loop linking intracellular acidification with higher acid-extrusion activity. This process resembles the response in myocytes described herein. The renal mechanism was proposed to involve the release of endothelin-1 (ET1), but acid-adaptation in myocytes was unaffected by the presence of the ET1 receptor antagonist bosentan (10 µM), arguing against the involvement of ET1 (*Figure 5F*).

The involvement of FAK1/Pyk2 in the link between chronic acidosis and NHE1 expression was tested pharmacologically. Neonatal myocytes were incubated at either pH 6.4 or 7.4 for 48 h in the presence or absence of PF-431396. Cells treated with PF-431396 had significantly reduced NHE1 expression, indicating that the inhibition of FAK-family kinases causes the pHi-regulatory apparatus to favour a more acidic set-point pHi (*Figure 5G*).

**Figure 6:**
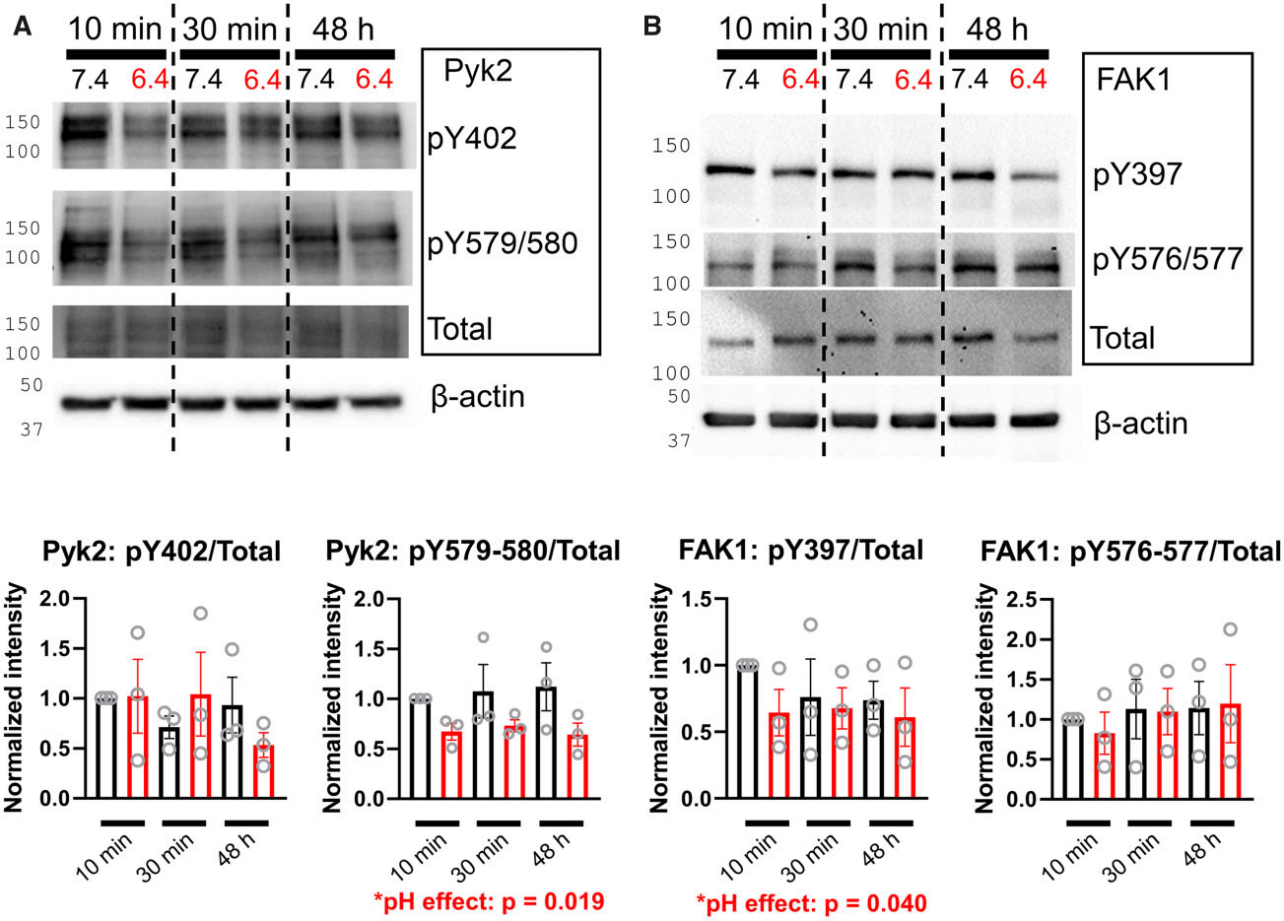
Acid triggers change in FAK-family phosphorylation. (*A*) Immunoblot for Pyk2 phosphorylation at Y402 and Y579/580, and total Pyk2 in lysates prepared after 10, 30 min, or 48 h of incubation at pH 6.4 (or time-matched for pH 7.4 as control). Exemplar blot from three biological repeats. (*B*) Immunoblot for FAK1 phosphorylation at Y397 and Y576/577, and total FAK1 in lysates prepared after 10, 30 min, or 48 h of incubation at pH 6.4 (or time-matched for pH 7.4 as control). Exemplar blot from three biological repeats. Densitometric quantification of blots from three independent isolations and treatment protocols. Two-way ANOVA tested for effect of pH.

To seek evidence that cardiomyocyte FAK1 and Pyk2 proteins respond post-translationally to an acidic stimulus, western blotting was performed on NRVMs exposed to acidic media (pH 6.4) for 10 min, 30 min, or 48 h, with appropriate time-matched controls (pH 7.4). An acid-evoked decrease in Y579/580 phosphorylation was detectable after 10 min of treatment, indicating a rapid-onset effect compatible with a trigger of acid-adaptation (*Figure 6A*). The same treatment protocols produced a more modest decrease in FAK1 phosphorylation at Y397 (*Figure 6B*). In summary, exposure to acidic conditions triggers, within minutes, a response in the FAK-family proteins Pyk2 and FAK1, the putative intracellular H^+^ sensors. Inhibiting FAK proteins pharmacologically ablates the acid-adaptation of pHi control, and decreases expression of NHE1.

## 4. Discussion

The urgency of maintaining a favourable pHi is demonstrated by the observation that cardiac physiology is highly pHi-sensitive^1–9^ and that the sarcolemma can generate H^+^-equivalent fluxes as large as tens of mM/min to correct pHi disturbances, the largest of all ionic fluxes recorded in cardiac cells.^10–14^ The regulatory prowess of acid–base transport has often led to the assumption that pHi is held firmly constant, unless instructed to change by neurohormonal factors. However, a major vulnerability in this control system relates to its sensitivity to pHe, a thermodynamic consequence of the transport cycle.^24,27,28^ Whereas pHi-sensitivity is obligatory for a homeostatic regulator of the internal milieu, the dependence on pHe should be considered a regulatory flaw because it inadvertently transfers the chemical vulnerability of the extracellular milieu onto the myocyte. This thermodynamic coupling would compromise pHi control—and hence cardiac function—unless a correction is implemented. This issue is particularly problematic in under-perfused niches emerging in disease states, such as the infarcted heart,^30,61^ or developmentally when vascular perfusion is undergoing maturation.^62–64^ Beyond the heart, a similar concern can apply to cerebral ischaemia, because neuronal function is also highly pH-sensitive.^65,66^ It has therefore been speculated that a secondary level of pHi control is necessary to correct for the coupling between pHe and pHi.

Herein, we describe a secondary layer of pHi homeostasis, which re-balances the expression of acid–base transporters, notably *Slc4a2* and *Slc9a1*, until their fluxes return steady-state pHi towards 7.1–7.2. This correction was observed *in vivo* in hearts following a period of recovery after cryo-infarction, a surgical intervention that produces a diffuse pattern of extracellular lactic acidosis, as well as in animals with supplemented systemic buffering that helps to maintain interstitial alkalinity near contracting myocytes. In the infarcted heart, myocytes adapt to the extracellular acidosis by increasing their acid-extrusion capacity through NHE1 and decreasing acid-loading through CBE. This response can restore a near-normal pHi in the beating heart, despite the persistence of the underlying extracellular acidosis. The adaptive correction was revealed as an overshoot in pHi after cells had been enzymically liberated and superfused at physiological pHe. Myocytes derived from the most under-perfused niches, determined by *in vivo* Hoechst staining, had the greatest degree of pHi remodelling. Consistent with a greater demand for acid-extrusion to raise pHi, NHE1 expression increased after cryo-infarction. In contrast, a reduction in demand for acid-extrusion in animals on oral bicarbonate resulted in NHE1 down-regulation, indicating that NHE1 expression is titrated on a needs basis. In these studies, NHE1 immunoreactivity was used as a readout of pHi control because anti-NHE1 antibodies are optimized for adequate densitometric quantification on western blot.

The response to acid-adaptation could be replicated *in vitro* using neonatal myocytes, which enabled further mechanistic studies using a high-throughput analysis pipeline. Transcriptomics identified three members of the ‘intracellular pH regulation’ ontology that respond to pHe: *Slc4a2, Slc9a1*, or *Slc4a7*. This result was confirmed at protein level for NHE1 and AE2 and functionally from sarcolemmal flux measurements. The ligand for the ‘acid sensor’ that triggers this transcriptional response was determined to be H^+^, rather than other acid–base proxies, such as CO_2_/HCO_3_^−^, the trigger for soluble adenylyl cyclase or RTPγ. The location of this H^+^ sensor was intracellular, rather than exofacial, which is desirable for a system designed to oversee pHi. The onset of the acid-adaptation response was slow, taking many hours, and its consequences on pHi control persisted after withdrawing the acid-treatment, at least in the time frame of measurements.

Although histone acetylation is known to be pH-sensitive through the catalytic responses of acetyl-transferase and deacetylase enzymes,^39^ pharmacological inhibition of HDACs had no effect on the acid-adaptation response. Transcriptomics profiling identified various kinases that affect the expression of *Slc4a2, Slc9a1*, or *Slc4a7.* A comparison of these against candidates for pH-sensors highlighted FAK-family non-receptor tyrosine kinases (FAK1/Pyk2)^41,42^ as possible transducers of acid-adaption. Indeed, acidic conditions evoked a change in FAK1 and Pyk2 phosphorylation, and FAK inhibition ablated the acid-adaptation response of pHi control by decreasing NHE1 expression. Drugs acting upstream to FAK had no effect on acid-adaptation outcomes, indicating FAK proteins as the entry point for H^+^ signals. Although protonation is an almost universal post-translational modification, only a small number of proteins, such as FAK-family kinases, have met the criteria for *bona fide* acid-sensors. Additional pH-sensing components along the acid-adaptation cascade cannot be excluded, as pharmacological FAK inhibition did not completely ablate the response measured in terms of pHi.

In summary, we have characterized how myocytes adapt their pHi-regulatory apparatus to acidic conditions, and thereby overcome the thermodynamic coupling between pHe and pHi, which would otherwise transfer the vulnerability of the external milieu onto untoward changes in pHi. The mechanism, operated by intracellular H^+^ sensors, also explains how the gene expression apparatus is instructed to titrate the correct level of acid-loaders and acid-extruders in order to attain physiological pHi. The operation of this feedback also explains how a cardiomyocyte determines what is deemed to be normal pHi. In future studies, it would be prudent to investigate the role of these pH-sensing mechanisms in other tissues, notably the brain, which is also susceptible to ischaemia.

## Supplementary material

Supplementary material is available at *Cardiovascular Research* online.

## Authors’ contributions

A.D.W., M.A.R., M.K.C., M.R., J.M., A.L., V.B., S.M., A.A.L., and C.C. performed the research. A.M., D.T., O.A.A., and Y.K.R. provided research materials. P.S. supervised the work. P.S. wrote the manuscript, and all authors contributed to the final draft.

## Funding

This work was supported by the British Heart Foundation Programme (RG/15/9/31534 to P.S. and RG/11/9/28921 to D.J.T.). This work was partially supported by the National Institutes of Health (NIH RO1 GM073857 to O.A.A. and Y.K.R.).

## Supplementary Material

cvab364_Supplementary_Data

## Data Availability

The data underlying this article are available in the article and in its online supplementary material. The data underlying this article will be shared upon reasonable request to the corresponding author. Translational perspective As a consequence of the inherent thermodynamic coupling between pHi/pHe, sustained changes to perfusion, such as those in coronary disease or development, would have deleterious effects on the internal acid–base milieu of myocytes and hence cardiac function, unless offset by a corrective process. Using *in vivo* and *in vitro* models of acidification, we characterize this adaptive process functionally, and describe how it is engaged to auto-regulate pHi. This additional layer of homeostatic oversight enables the myocardium to operate within its optimal pHi-range, even at times when vascular perfusion is failing to maintain chemical constancy of the interstitial fluid.

## References

[1] Garciarena CD , Youm JB, Swietach P, Vaughan-Jones RD. H(+)activated Na(+) influx in the ventricular myocyte couples Ca(2)(+)-signalling to intracellular pH. J Mol Cell Cardiol 2013;61:51–59. 23602948 10.1016/j.yjmcc.2013.04.008

[2] Vaughan-Jones RD , Spitzer KW, Swietach P. Intracellular pH regulation in heart. J Mol Cell Cardiol 2009;46:318–331. 19041875 10.1016/j.yjmcc.2008.10.024

[3] Fabiato A , Fabiato F. Effects of pH on the myofilaments and the sarcoplasmic reticulum of skinned cells from cardiace and skeletal muscles. J Physiol 1978;276:233–255. 25957 10.1113/jphysiol.1978.sp012231 PMC1282422

[4] Bountra C , Vaughan-Jones RD. Effect of intracellular and extracellular pH on contraction in isolated, mammalian cardiac muscle. J Physiol 1989;418:163–187. 2621616 10.1113/jphysiol.1989.sp017833 PMC1189964

[5] Orchard CH , Kentish JC. Effects of changes of pH on the contractile function of cardiac muscle. Am J Physiol 1990;258:C967–C981. 2193525 10.1152/ajpcell.1990.258.6.C967

[6] Harrison SM , Frampton JE, McCall E, Boyett MR, Orchard CH. Contraction and intracellular Ca2+, Na+, and H+ during acidosis in rat ventricular myocytes. Am J Physiol 1992;262:C348–C357. 1539627 10.1152/ajpcell.1992.262.2.C348

[7] Allen DG , Orchard CH. The effects of changes of pH on intracellular calcium transients in mammalian cardiac muscle. J Physiol 1983;335:555–567. 6410050 10.1113/jphysiol.1983.sp014550 PMC1197369

[8] Choi HS , Trafford AW, Orchard CH, Eisner DA. The effect of acidosis on systolic Ca2+ and sarcoplasmic reticulum calcium content in isolated rat ventricular myocytes. J Physiol 2000;529 (pt. 3):661–668. 11118496 10.1111/j.1469-7793.2000.00661.x PMC2270229

[9] Orchard CH , Cingolani HE. Acidosis and arrhythmias in cardiac muscle. Cardiovasc Res 1994;28:1312–1319. 7954638 10.1093/cvr/28.9.1312

[10] Leem CH , Lagadic-Gossmann D, Vaughan-Jones RD. Characterization of intracellular pH regulation in the guinea-pig ventricular myocyte. J Physiol 1999;517:159–180. 10226157 10.1111/j.1469-7793.1999.0159z.x PMC2269328

[11] Sun B , Leem CH, Vaughan-Jones RD. Novel chloride-dependent acid loader in the guinea-pig ventricular myocyte: part of a dual acid-loading mechanism. J Physiol 1996;495:65–82. 8866352 10.1113/jphysiol.1996.sp021574 PMC1160725

[12] Poole RC , Halestrap AP, Price SJ, Levi AJ. The kinetics of transport of lactate and pyruvate into isolated cardiac myocytes from guinea pig. Kinetic evidence for the presence of a carrier distinct from that in erythrocytes and hepatocytes. Biochem J 1989;264:409–418. 2604725 10.1042/bj2640409 PMC1133596

[13] Lagadic-Gossmann D , Buckler KJ, Vaughan-Jones RD. Role of bicarbonate in pH recovery from intracellular acidosis in the guinea-pig ventricular myocyte. J Physiol 1992;458:361–384. 1302269 10.1113/jphysiol.1992.sp019422 PMC1175160

[14] Xu P , Spitzer KW. Na-independent Cl(-)-HCO3- exchange mediates recovery of pHi from alkalosis in guinea pig ventricular myocytes. Am J Physiol 1994;267:H85–H91. 8048611 10.1152/ajpheart.1994.267.1.H85

[15] Orlowski J , Grinstein S. Diversity of the mammalian sodium/proton exchanger SLC9 gene family. Pflugers Arch 2004;447:549–565. 12845533 10.1007/s00424-003-1110-3

[16] Sardet C , Franchi A, Pouyssegur J. Molecular cloning, primary structure, and expression of the human growth factor-activatable Na+/H+ antiporter. Cell 1989;56:271–280. 2536298 10.1016/0092-8674(89)90901-x

[17] Choi I , Romero MF, Khandoudi N, Bril A, Boron WF. Cloning and characterization of a human electrogenic Na+-HCO-3 cotransporter isoform (hhNBC). Am J Physiol 1999;276:C576–C584. 10069984 10.1152/ajpcell.1999.276.3.C576

[18] Romero MF , Fulton CM, Boron WF. The SLC4 family of HCO 3 - transporters. Pflugers Arch 2004;447:495–509. 14722772 10.1007/s00424-003-1180-2

[19] Yamamoto T , Shirayama T, Sakatani T, Takahashi T, Tanaka H, Takamatsu T, Spitzer KW, Matsubara H. Enhanced activity of ventricular Na+-HCO3- cotransport in pressure overload hypertrophy. Am J Physiol Heart Circ Physiol 2007;293:H1254–H1264. 17416604 10.1152/ajpheart.00964.2006

[20] Virkki LV , Wilson DA, Vaughan-Jones RD, Boron WF. Functional characterization of human NBC4 as an electrogenic Na+-HCO cotransporter (NBCe2). Am J Physiol Cell Physiol 2002;282:C1278–C1289. 11997242 10.1152/ajpcell.00589.2001

[21] Grichtchenko II , Choi I, Zhong X, Bray-Ward P, Russell JM, Boron WF. Cloning, characterization, and chromosomal mapping of a human electroneutral Na(+)-driven Cl-HCO3 exchanger. J Biol Chem 2001;276:8358–8363. 11133997 10.1074/jbc.C000716200

[22] Alper SL . Molecular physiology of SLC4 anion exchangers. Exp Physiol 2006;91:153–161. 16239253 10.1113/expphysiol.2005.031765

[23] Richards SM , Jaconi ME, Vassort G, Puceat M. A spliced variant of AE1 gene encodes a truncated form of Band 3 in heart: the predominant anion exchanger in ventricular myocytes. J Cell Sci 1999;112:1519–1528. 10212146 10.1242/jcs.112.10.1519

[24] Niederer SA , Swietach P, Wilson DA, Smith NP, Vaughan-Jones RD. Measuring and modeling chloride-hydroxyl exchange in the Guinea-pig ventricular myocyte. Biophys J 2008;94:2385–2403. 18055536 10.1529/biophysj.107.118885 PMC2257879

[25] Alvarez BV , Kieller DM, Quon AL, Markovich D, Casey JR. Slc26a6: a cardiac chloride-hydroxyl exchanger and predominant chloride-bicarbonate exchanger of the mouse heart. J Physiol 2004;561:721–734. 15498800 10.1113/jphysiol.2004.077339 PMC1665392

[26] Shcheynikov N , Wang Y, Park M, Ko SB, Dorwart M, Naruse S, Thomas PJ, Muallem S. Coupling modes and stoichiometry of Cl-/HCO3- exchange by slc26a3 and slc26a6. J Gen Physiol 2006;127:511–524. 16606687 10.1085/jgp.200509392 PMC2151520

[27] Vaughan-Jones RD , Wu ML. Extracellular H+ inactivation of Na(+)-H+ exchange in the sheep cardiac Purkinje fibre. J Physiol 1990;428:441–466. 2172524 10.1113/jphysiol.1990.sp018221 PMC1181656

[28] Jean T , Frelin C, Vigne P, Barbry P, Lazdunski M. Biochemical properties of the Na+/H+ exchange system in rat brain synaptosomes. Interdependence of internal and external pH control of the exchange activity. J Biol Chem 1985;260:9678–9684. 2991259

[29] Steenbergen C , Deleeuw G, Rich T, Williamson JR. Effects of acidosis and ischemia on contractility and intracellular pH of rat heart. Circ Res 1977;41:849–858. 21759 10.1161/01.res.41.6.849

[30] Garlick PB , Radda GK, Seeley PJ. Studies of acidosis in the ischaemic heart by phosphorus nuclear magnetic resonance. Biochem J 1979;184:547–554. 44193 10.1042/bj1840547 PMC1161836

[31] Yan GX , Kleber AG. Changes in extracellular and intracellular pH in ischemic rabbit papillary muscle. Circ Res 1992;71:460–470. 1628400 10.1161/01.res.71.2.460

[32] Sardet C , Counillon L, Franchi A, Pouyssegur J. Growth factors induce phosphorylation of the Na+/H+ antiporter, glycoprotein of 110 kD. Science 1990;247:723–726. 2154036 10.1126/science.2154036

[33] Moolenaar WH , Tsien RY, van der Saag PT, de Laat SW. Na+/H+ exchange and cytoplasmic pH in the action of growth factors in human fibroblasts. Nature 1983;304:645–648. 6410286 10.1038/304645a0

[34] Lagadic-Gossmann D , Vaughan-Jones RD. Coupling of dual acid extrusion in the guinea-pig isolated ventricular myocyte to alpha 1- and beta-adrenoceptors. J Physiol 1993;464:49–73. 7901399 10.1113/jphysiol.1993.sp019624 PMC1175375

[35] Puceat M , Clement-Chomienne O, Terzic A, Vassort G. Alpha 1-adrenoceptor and purinoceptor agonists modulate Na-H antiport in single cardiac cells. Am J Physiol 1993;264:H310–H319. 8095373 10.1152/ajpheart.1993.264.2.H310

[36] Matsui H , Barry WH, Livsey C, Spitzer KW. Angiotensin II stimulates sodium-hydrogen exchange in adult rabbit ventricular myocytes. Cardiovasc Res 1995;29:215–221. 7736498

[37] Gunasegaram S , Haworth RS, Hearse DJ, Avkiran M. Regulation of sarcolemmal Na(+)/H(+) exchanger activity by angiotensin II in adult rat ventricular myocytes: opposing actions via AT(1) versus AT(2) receptors. Circ Res 1999;85:919–930. 10559139 10.1161/01.res.85.10.919

[38] Richards MA , Simon JN, Ma R, Loonat AA, Crabtree MJ, Paterson DJ, Fahlman RP, Casadei B, Fliegel L, Swietach P. Nitric oxide modulates cardiomyocyte pH control through a biphasic effect on sodium/hydrogen exchanger-1. Cardiovasc Res 2020;116:1958–1971. 31742355 10.1093/cvr/cvz311 PMC7567331

[39] McBrian MA , Behbahan IS, Ferrari R, Su T, Huang TW, Li K, Hong CS, Christofk HR, Vogelauer M, Seligson DB, Kurdistani SK. Histone acetylation regulates intracellular pH. Mol Cell 2013;49:310–321. 23201122 10.1016/j.molcel.2012.10.025 PMC3893119

[40] Choi CH , Webb BA, Chimenti MS, Jacobson MP, Barber DL. pH sensing by FAK-His58 regulates focal adhesion remodeling. J Cell Biol 2013;202:849–859. 24043700 10.1083/jcb.201302131 PMC3776353

[41] Preisig PA . The acid-activated signaling pathway: starting with Pyk2 and ending with increased NHE3 activity. Kidney Int 2007;72:1324–1329. 17882150 10.1038/sj.ki.5002543

[42] Li S , Sato S, Yang X, Preisig PA, Alpern RJ. Pyk2 activation is integral to acid stimulation of sodium/hydrogen exchanger 3. J Clin Invest 2004;114:1782–1789. 15599403 10.1172/JCI18046 PMC535061

[43] Chen Y , Cann MJ, Litvin TN, Iourgenko V, Sinclair ML, Levin LR, Buck J. Soluble adenylyl cyclase as an evolutionarily conserved bicarbonate sensor. Science 2000;289:625–628. 10915626 10.1126/science.289.5479.625

[44] Tomura H , Mogi C, Sato K, Okajima F. Proton-sensing and lysolipid-sensitive G-protein-coupled receptors: a novel type of multi-functional receptors. Cell Signal 2005;17:1466–1476. 16014326 10.1016/j.cellsig.2005.06.002

[45] Ludwig MG , Vanek M, Guerini D, Gasser JA, Jones CE, Junker U, Hofstetter H, Wolf RM, Seuwen K. Proton-sensing G-protein-coupled receptors. Nature 2003;425:93–98. 12955148 10.1038/nature01905

[46] Lewis AJM , Miller JJ, Lau AZ, Curtis MK, Rider OJ, Choudhury RP, Neubauer S, Cunningham CH, Carr CA, Tyler DJ. Noninvasive immunometabolic cardiac inflammation imaging using hyperpolarized magnetic resonance. Circ Res 2018;122:1084–1093. 29440071 10.1161/CIRCRESAHA.117.312535 PMC5908252

[47] Ciulla MM , Paliotti R, Ferrero S, Braidotti P, Esposito A, Gianelli U, Busca G, Cioffi U, Bulfamante G, Magrini F. Left ventricular remodeling after experimental myocardial cryoinjury in rats. J Surg Res 2004;116:91–97. 14732353 10.1016/j.jss.2003.08.238

[48] Sosunov EA , Anyukhovsky EP, Sosunov AA, Moshnikova A, Wijesinghe D, Engelman DM, Reshetnyak YK, Andreev OA. pH (low) insertion peptide (pHLIP) targets ischemic myocardium. Proc Natl Acad Sci USA 2013;110:82–86. 23248283 10.1073/pnas.1220038110 PMC3538213

[49] Ford KL , Moorhouse EL, Bortolozzi M, Richards MA, Swietach P, Vaughan-Jones RD. Regional acidosis locally inhibits but remotely stimulates Ca2+ waves in ventricular myocytes. Cardiovasc Res 2017;113:984–995. 28339694 10.1093/cvr/cvx033 PMC5852542

[50] Chung YJ , Luo A, Park KC, Loonat AA, Lakhal-Littleton S, Robbins PA, Swietach P. Iron-deficiency anemia reduces cardiac contraction by downregulating RyR2 channels and suppressing SERCA pump activity. JCI Insight 2019;4:e125618. 10.1172/jci.insight.125618 PMC6483648 30779710

[51] Loonat AA , Curtis MK, Richards MA, Nunez-Alonso G, Michl J, Swietach P. A high-throughput ratiometric method for imaging hypertrophic growth in cultured primary cardiac myocytes. J Mol Cell Cardiol 2019;130:184–196. 30986378 10.1016/j.yjmcc.2019.04.001 PMC6520438

[52] Zoccarato A , Surdo NC, Aronsen JM, Fields LA, Mancuso L, Dodoni G, Stangherlin A, Livie C, Jiang H, Sin YY, Gesellchen F, Terrin A, Baillie GS, Nicklin SA, Graham D, Szabo-Fresnais N, Krall J, Vandeput F, Movsesian M, Furlan L, Corsetti V, Hamilton G, Lefkimmiatis K, Sjaastad I, Zaccolo M. Cardiac hypertrophy is inhibited by a local pool of cAMP regulated by phosphodiesterase 2. Circ Res 2015;117:707–719. 26243800 10.1161/CIRCRESAHA.114.305892

[53] Michl J , Park KC, Swietach P. Evidence-based guidelines for controlling pH in mammalian live-cell culture systems. Commun Biol 2019;2:144. 31044169 10.1038/s42003-019-0393-7 PMC6486606

[54] Sikkel MB , Francis DP, Howard J, Gordon F, Rowlands C, Peters NS, Lyon AR, Harding SE, MacLeod KT. Hierarchical statistical techniques are necessary to draw reliable conclusions from analysis of isolated cardiomyocyte studies. Cardiovasc Res 2017;113:1743–1752. 29016722 10.1093/cvr/cvx151 PMC5852514

[55] Tomek J , Hao G, Tomkova M, Lewis A, Carr C, Paterson DJ, Rodriguez B, Bub G, Herring N. beta-Adrenergic receptor stimulation and alternans in the border zone of a healed infarct: an *ex vivo* study and computational investigation of arrhythmogenesis. Front Physiol 2019;10:350. 30984029 10.3389/fphys.2019.00350 PMC6450465

[56] Robey IF , Baggett BK, Kirkpatrick ND, Roe DJ, Dosescu J, Sloane BF, Hashim AI, Morse DL, Raghunand N, Gatenby RA, Gillies RJ. Bicarbonate increases tumor pH and inhibits spontaneous metastases. Cancer Res 2009;69:2260–2268. 19276390 10.1158/0008-5472.CAN-07-5575 PMC2834485

[57] van Hasselt JGC , Rahman R, Hansen J, Stern A, Shim JV, Xiong Y, Pickard A, Jayaraman G, Hu B, Mahajan M, Gallo JM, Goldfarb J, Sobie EA, Birtwistle MR, Schlessinger A, Azeloglu EU, Iyengar R. Transcriptomic profiling of human cardiac cells predicts protein kinase inhibitor-associated cardiotoxicity. Nat Commun 2020;11:4809. 32968055 10.1038/s41467-020-18396-7 PMC7511315

[58] Takeishi Y . Pivotal roles of regulating the proline-rich tyrosine kinase 2 (PYK2) signaling in cardiac function and remodeling. J Mol Cell Cardiol 2014;74:295–296. 24956220 10.1016/j.yjmcc.2014.06.005

[59] Takahashi T . Pyk2/CAKbeta signaling: a novel Ca(2+)-dependent pathway leading to cardiac hypertrophy. J Mol Cell Cardiol 2004;36:791–793. 15158119 10.1016/j.yjmcc.2004.03.011

[60] Melendez J , Welch S, Schaefer E, Moravec CS, Avraham S, Avraham H, Sussman MA. Activation of pyk2/related focal adhesion tyrosine kinase and focal adhesion kinase in cardiac remodeling. J Biol Chem 2002;277:45203–45210. 12228222 10.1074/jbc.M204886200

[61] Elliott AC , Smith GL, Eisner DA, Allen DG. Metabolic changes during ischaemia and their role in contractile failure in isolated ferret hearts. J Physiol 1992;454:467–490. 1474498 10.1113/jphysiol.1992.sp019274 PMC1175615

[62] Lupu IE , De Val S, Smart N. Coronary vessel formation in development and disease: mechanisms and insights for therapy. Nat Rev Cardiol 2020;17:790–806. 32587347 10.1038/s41569-020-0400-1

[63] Puente BN , Kimura W, Muralidhar SA, Moon J, Amatruda JF, Phelps KL, Grinsfelder D, Rothermel BA, Chen R, Garcia JA, Santos CX, Thet S, Mori E, Kinter MT, Rindler PM, Zacchigna S, Mukherjee S, Chen DJ, Mahmoud AI, Giacca M, Rabinovitch PS, Aroumougame A, Shah AM, Szweda LI, Sadek HA. The oxygen-rich postnatal environment induces cardiomyocyte cell-cycle arrest through DNA damage response. Cell 2014;157:565–579. 24766806 10.1016/j.cell.2014.03.032 PMC4104514

[64] Neary MT , Ng KE, Ludtmann MH, Hall AR, Piotrowska I, Ong SB, Hausenloy DJ, Mohun TJ, Abramov AY, Breckenridge RA. Hypoxia signaling controls postnatal changes in cardiac mitochondrial morphology and function. J Mol Cell Cardiol 2014;74:340–352. 24984146 10.1016/j.yjmcc.2014.06.013 PMC4121533

[65] Chesler M , Kaila K. Modulation of pH by neuronal activity. Trends Neurosci 1992;15:396–402. 1279865 10.1016/0166-2236(92)90191-a

[66] Nedergaard M , Kraig RP, Tanabe J, Pulsinelli WA. Dynamics of interstitial and intracellular pH in evolving brain infarct. Am J Physiol 1991;260:R581–R588. 2001008 10.1152/ajpregu.1991.260.3.R581 PMC3062631

