# Supplementary material for: Acidic environments trigger intracellular H+-sensing FAK proteins to re-balance sarcolemmal acid–base transporters and auto-regulate cardiomyocyte pH
Source: Cardiovasc Res. 2021 Dec 13;118(14):2946–59. doi: 10.1093/cvr/cvab364 (PMC9648823; doi:10.1093/cvr/cvab364)
Supplement: cvab364_Supplementary_Data [file cvab364_supplementary_data.pdf]

Acidic environments trigger intracellular H^+-sensing FAK proteins to re-balance sarcolemmal acid-base transporters and auto-regulate cardiomyocyte pH

Abigail D. Wilson, Mark A. Richards, M. Kate Curtis, Mala Rohling, Stefania Monterisi, Aminah A. Loonat, Jack Miller, Vicky Ball, Andrew Lewis, Damian Tyler, Anna Moshnikova, Oleg A. Andreev, Yana K. Reshetnyak, Carolyn Carr, Pawel Swietach

SUPPLEMENTARY METHODS AND FIGURES

SUPPLEMENTARY METHODS

pHLIP synthesis. Peptide concentration was calculated by absorbance at 280 nm in methanol, using extinction coefficient ε_{280} = 12,660 M^{-1} cm^{-1}. Cy5.5-maleimide concentration was calculated by absorbance at 684 nm in methanol, using extinction coefficient ε_{800} = 198,000 M^{-1} cm^{-1}. Cy5.5-maleimide was mixed with the peptide at the molar ratio of 1:1. 100 mM sodium phosphate buffer, pH 7.2, containing 150 mM NaCl was saturated with argon and added to the reaction mixture (1/20 of total volume). Reaction mixture was incubated at room temperature for 2 hours and the reaction progress was monitored by the analytical reverse phase HPLC (Zorbax SB-C18 column 4.6 x 250 mm, 5 µm; Agilent Technologies; the gradient of binary solvent system using water and acetonitrile with 0.05% TFA for 20-80% over 30 min). Cy5.5-pHLIP was purified by the reverse phase HPLC (Zorbax SB-C18 columns 9.4 x 250 mm, 5 µm; Agilent Technologies, the same gradient, over 40 min), lyophilized and characterized by SELDI-TOF mass spectrometry.

^{13}C magnetic resonance imaging. After cryo-injury or sham surgery as described above, rats were studied in the early absorptive (fed) state between 0600 and 1300. Animals were anaesthetized by isoflurane in oxygen (3% for induction, 2% for maintenance), intravenous access gained using a 27 G tail-vein cannula and ECG electrodes connected to a home-built ECG triggering system[1,2] inserted into the lateral aspects of the thorax before being positioned within a homeothermic imaging cradle in a preclinical 7 T horizontal bore MRI system (Varian DDR2). Hyperpolarized magnetic resonance imaging was performed using an actively detuned transmit/surface receive setup consisting of a 72 mm dual-tuned ^{1}H/^{13}C proton/carbon birdcage volume coil with a 40 mm two-channel ^{13}C surface receive array with an integrated preamp (Rapid Biomedical GMBH, Rimpar, Germany). Correct positioning was confirmed by the acquisition of an axial proton localisers (3D, proton-density weighted T1 images) followed by the acquisition of cardiac-gated segmented spoiled gradient echo images in the short axis (TR: 4.6 ms; TE: 1.534 ms; 64 × 64 mm FOV; 96 × 96 matrix). A cardiac-specific automatic shimming algorithm was subsequently employed to reduce the proton linewidth to <100 Hz from the region of interest. As described previously[3], hyperpolarised pyruvate was prepared using 40 mg [1-13C]pyruvic acid, doped with 15 mM OX063 trityl radical and trace Dotarem gadolinium chelate in a prototype hyperpolariser operating at 3.35 T and 1.4 K. Dissolution was rapidly performed in superheated EDTA buffer, and 2 mL of the resulting 80 mM pyruvate solution (with a liquid state nuclear polarization of 30–40 %) was injected manually over 20 s via a preplaced tail vein catheter. Hyperpolarised 13C MR images of pyruvate and the downstream production of 13C-labelled lactate and bicarbonate were then acquired through the use of a custom-designed spectral-spatial echo-planar imaging sequence that provided 3-dimensional cardiac metabolite mapping with a spatial resolution of 2×2×3.8 mm^3 and temporal resolution of 1.8 seconds, acquired and reconstructed as described in detail previously[4,5].

^{13}C magnetic resonance spectroscopy. Rats were anaesthetized and positioned within the 7 T MRI system as described above. Correct positioning was confirmed by the acquisition of an axial proton FLASH image (T_E/T_R, 1.17/2.33 ms; matrix size, 64 x 64; FOV, 60 x 60 mm^2; slice thickness, 2.5 mm; excitation flip angle, 15°). An ECG-gated shim was used to reduce proton linewidth to ~120 Hz. One mL of hyperpolarized pyruvate, preparation described above, was injected over 10 s, followed by a 0.05 mL flush of heparinized saline to clear the delivery line. Sixty individual ECG-gated ^{13}C MR pulse-acquire cardiac spectra were acquired over 1 min following injection and subsequently analysed using the AMARES algorithm in the jMRUI software package (Version 4.0)[6]. The ratio of hyperpolarised HCO_3^-/CO_2 was used in the Henderson-Hasselbalch equation to estimate pHi[7].

Quantitative PCR. Total RNA was extracted from NRVMs using TRIzol (Invitrogen), and extracted from heart tissue using RNeasy Fibrous Tissue Mini Kit (Qiagen). RNA samples

were reverse transcribed using SuperScript IV Reverse Transcriptase (Thermo Fisher Scientific) according to manufacturer's instructions. Quantitative real-time PCR was performed in duplex reactions with housekeeping genes using TaqMan probes (Applied Biosystems). Reactions were set up using TaqMan Fast Universal PCR Master Mix and assays were performed in a ViiA 7 Real-Time PCR System (Applied Biosystems).

RNAseq. RNAseq data were analyzed by the DESeq2 package in R.

SUPPLEMENTARY TABLE

Inhibitor	*SLC4A2*	*SLC9A1*	*SLC4A7*
Sorafenib	-1.1067	+1.2784	+0.73166
Sorafenib	n.s.	+0.92838	+0.59689
Sorafenib	n.s.	+0.80136	n.s.
Nilotinib	-1.487	n.s.	-0.62711
Nilotinib	-1.0596	n.s.	-0.91798
Nilotinib	n.s.	+1.0829	n.s.
Regorafenib	n.s.	+1.0422	n.s.
Regorafenib	n.s.	+0.79755	n.s.
Vemurafenib	n.s.	+1.3878	n.s.
Trametinib	+0.7003	n.s.	-0.49843
Cabozantinib	n.s.	-1.2621	n.s.
Dabrafenib	n.s.	-1.3774	n.s.
Pazopanib	+0.92984	n.s.	+0.88393

Table S1: Analysis of transcriptional datasets from van Hasselt et al[8]. Table shows log2 fold-change in expression of genes of interest (*SLC9A1, SLC4A2, SLC4A7*) in human cardiac cells in response to kinase inhibitors. Only significant chnages are listed (FDR<0.05). Colour-coding proportional to fold-change. N.s. = not significant. Data retrieved from GEO accession # GSE146096.

SUPPLEMENTARY FIGURES

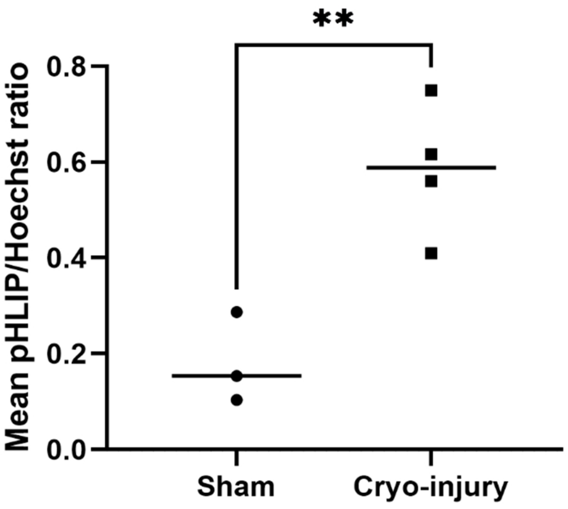

Figure S1: Quantification of pHLIP/Hoechst ratio across myocardium of rat hearts, five weeks after cryo-infarction or sham-surgery. T-test: P<0.01.

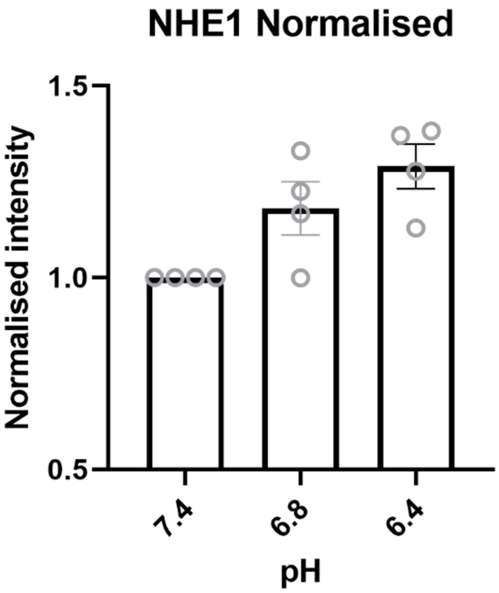

Figure S2: Densitometric quantification of NHE1 in neonatal ventricular myocytes adapted to pH 6.4, 6.9 or 7.4 for 48 h. Experiments were repeated on four independent myocyte isolations from 10-12 pups each. Measurements of NHE1/actin ratio were normalized to values for 7.4. Correlation with pH is significant (Pearson's test: P=0.016).

UNEDITED BLOTS

Fig. 2E_Top

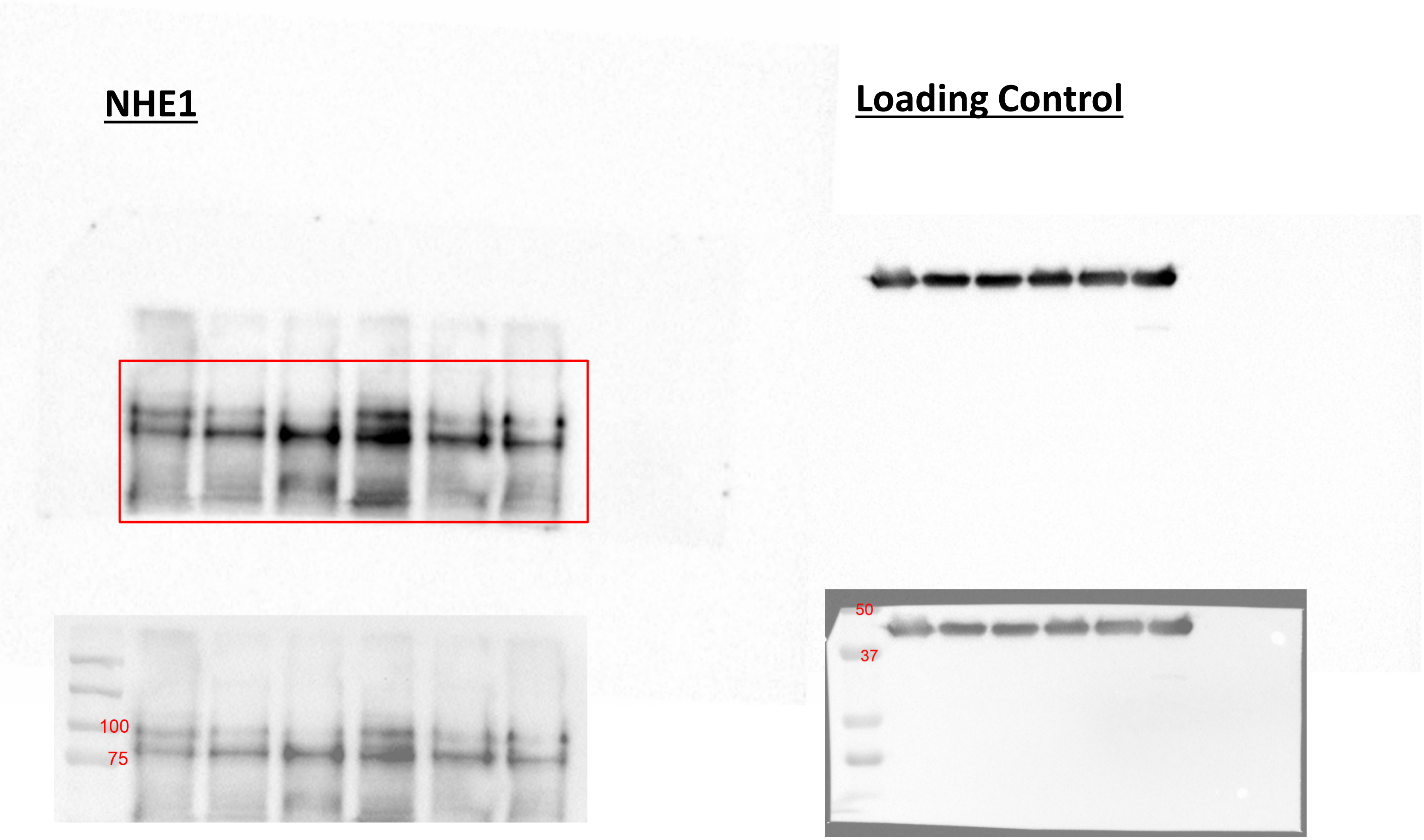

Uncropped gel

MW marker superimposed

Fig. 2E_Bottom

NHE1

Loading Control

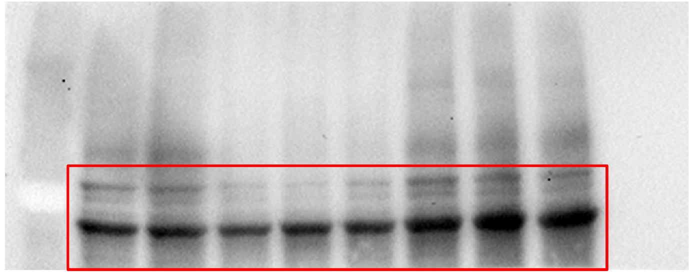

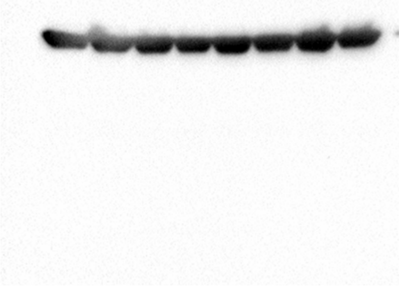

Uncropped gel

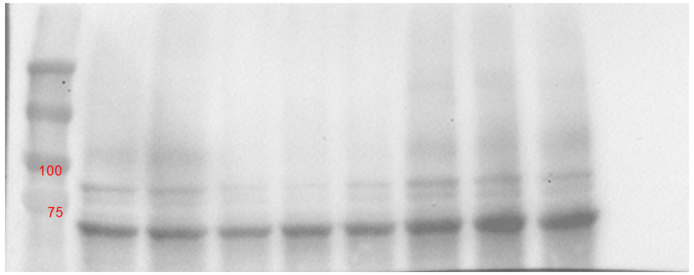

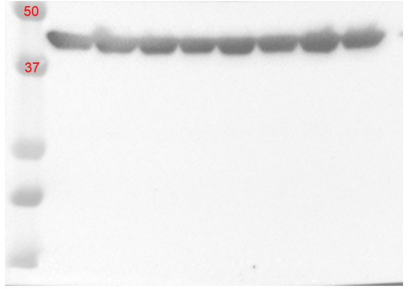

MW marker superimposed

Fig. 2F

NHE1

Loading Control

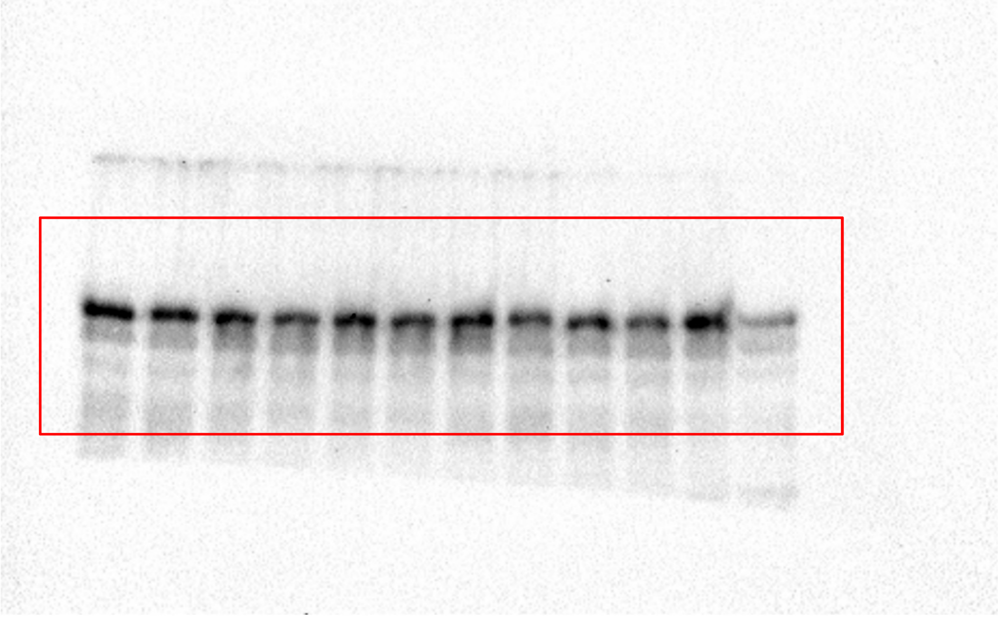

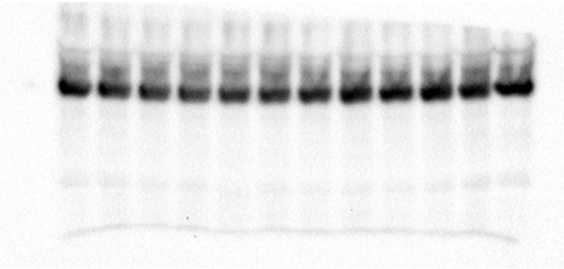

Uncropped gel

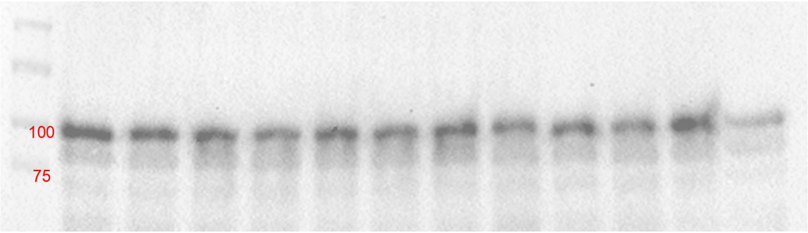

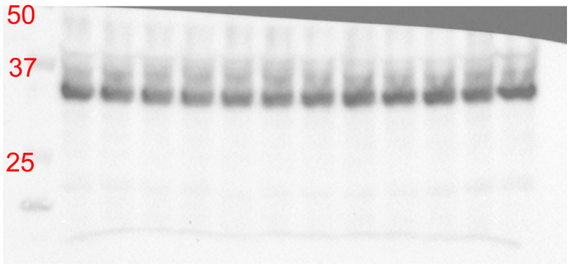

MW marker superimposed

Fig. 3I_Top

NHE1

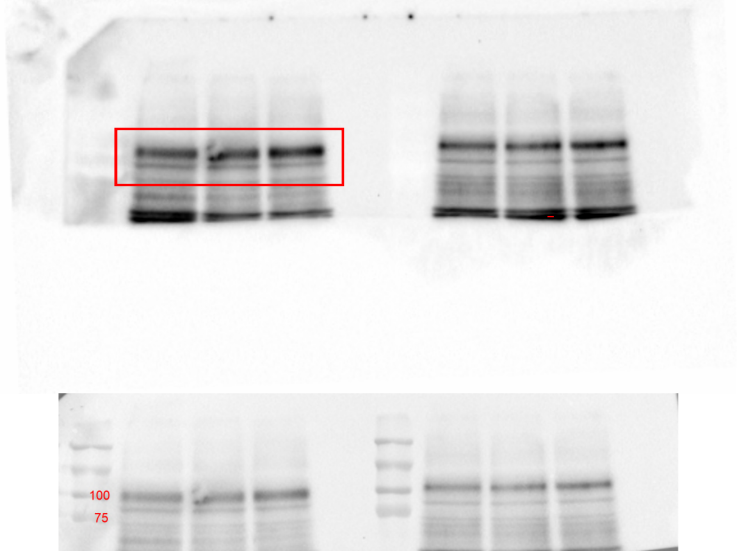

Loading Control

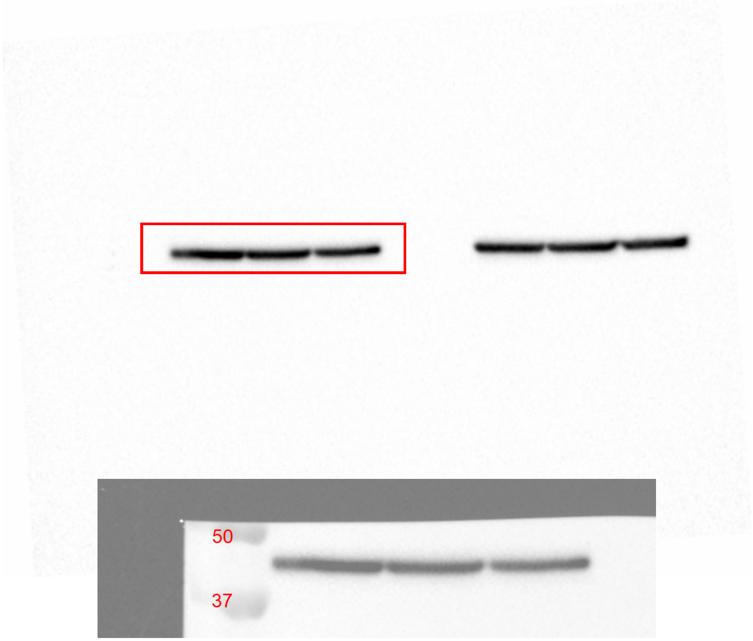

Uncropped gel

MW marker superimposed

Fig. 3I_Bottom

AE2

Loading Control

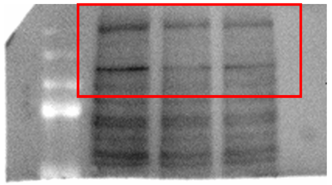

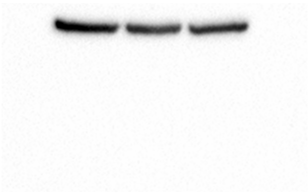

Uncropped gel

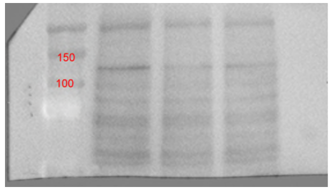

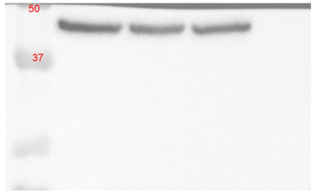

MW marker superimposed

Fig. 5G

<u>NHE1</u>

<u>Loading Control</u>

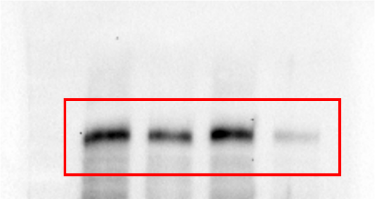

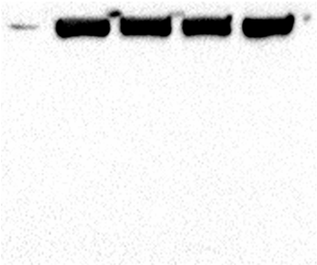

Uncropped gel

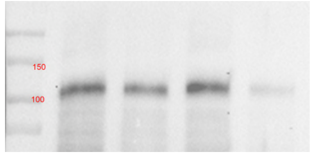

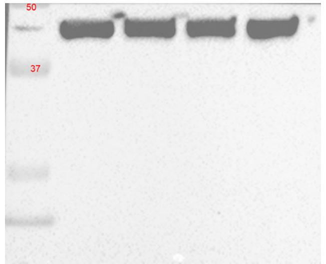

MW marker superimposed

Fig. 6A

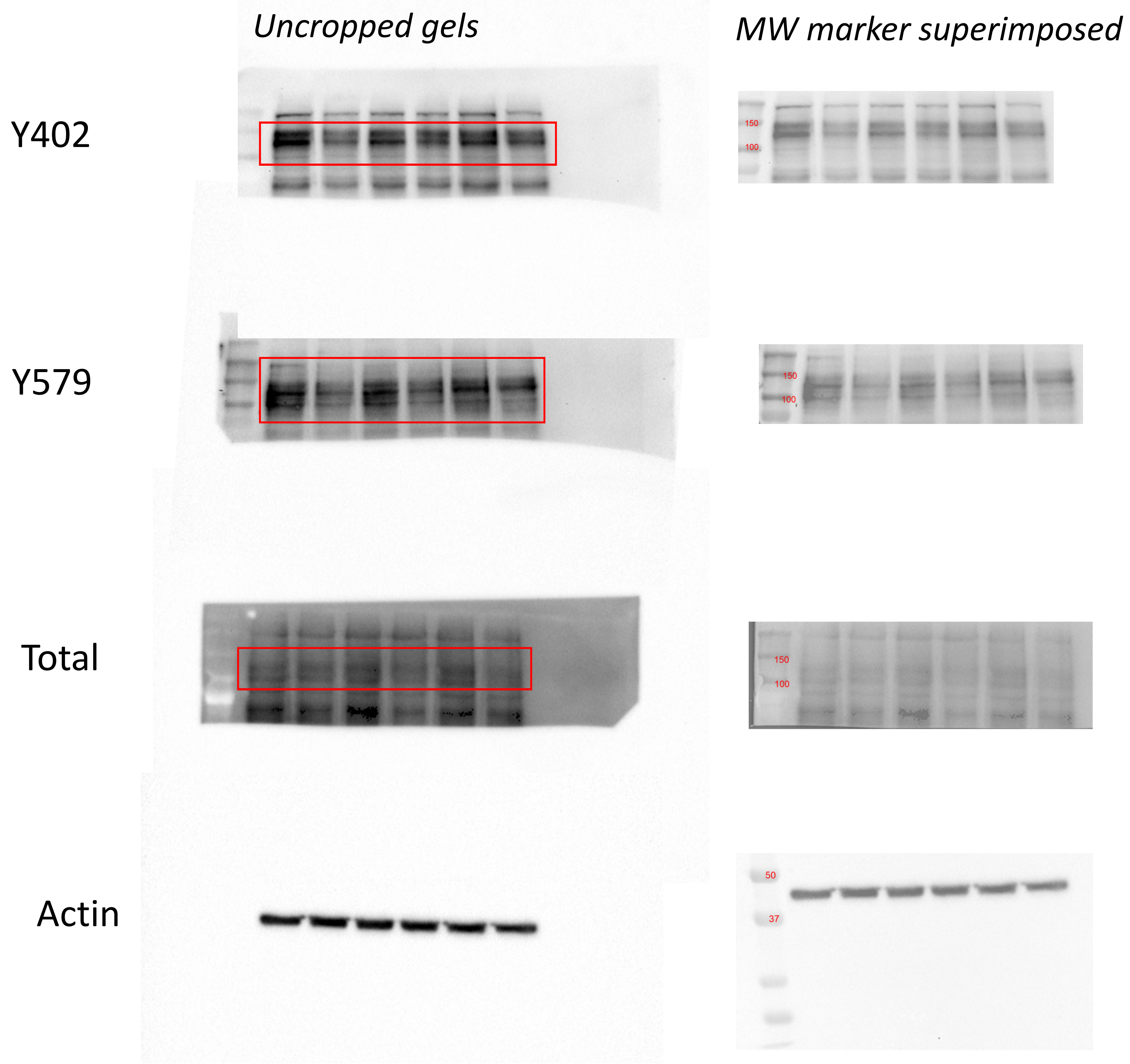

Uncropped gels
MW marker superimposed
Y402
150
100
Y579
150
100
Total
150
100
Actin
50
37

Fig. 6B

Uncropped gels

MW marker superimposed

Y397

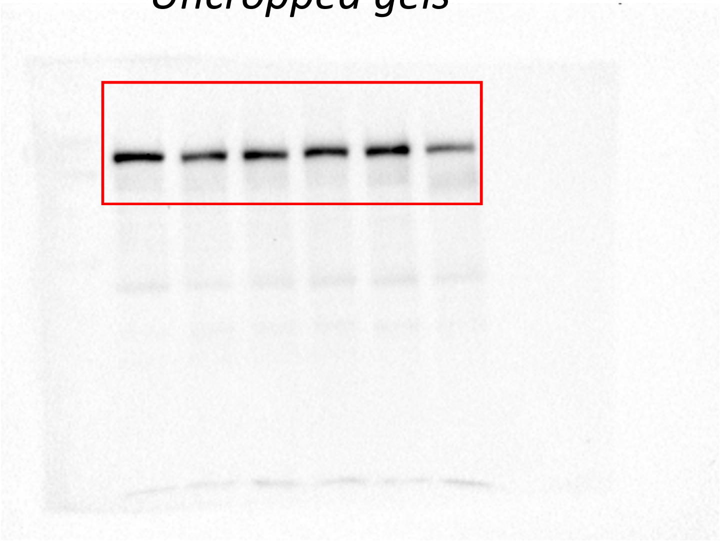

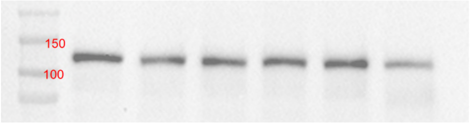

Y579

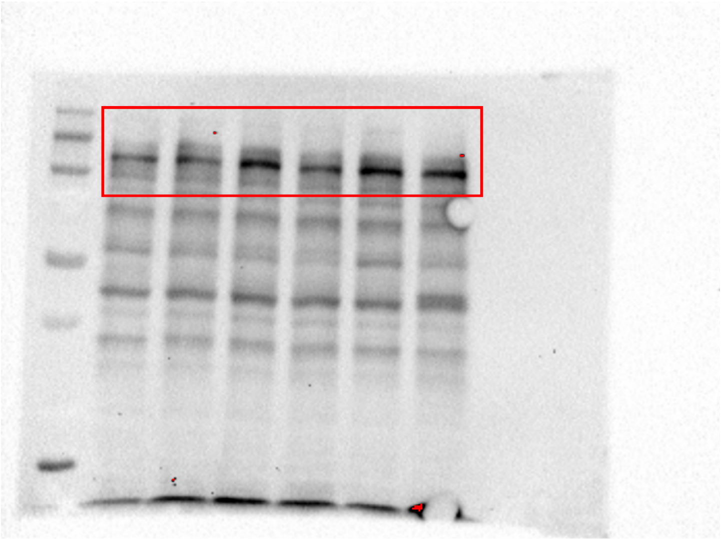

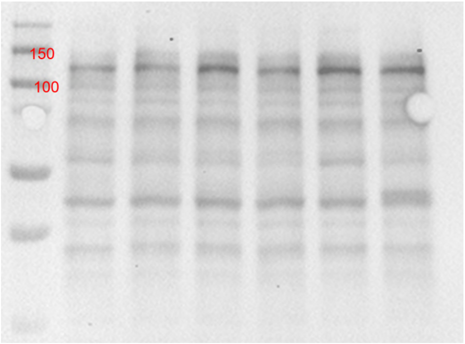

Cont. on next slide

Fig. 6B_Continued

Uncropped gels

MW marker superimposed

Total

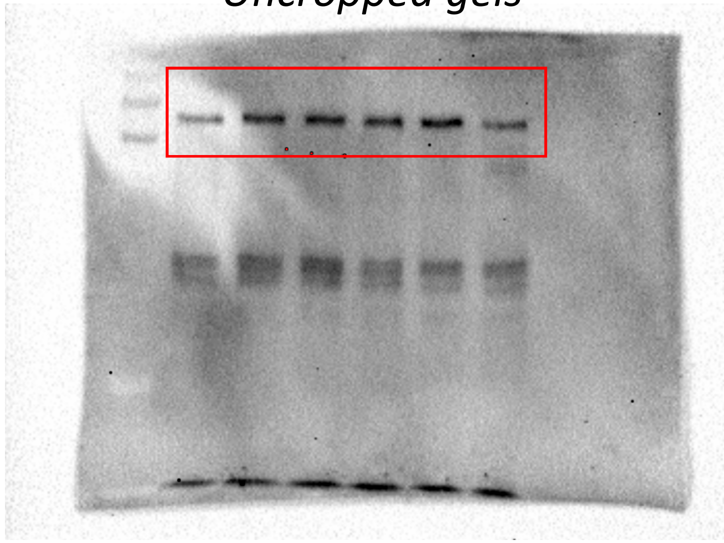

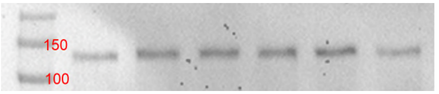

Actin

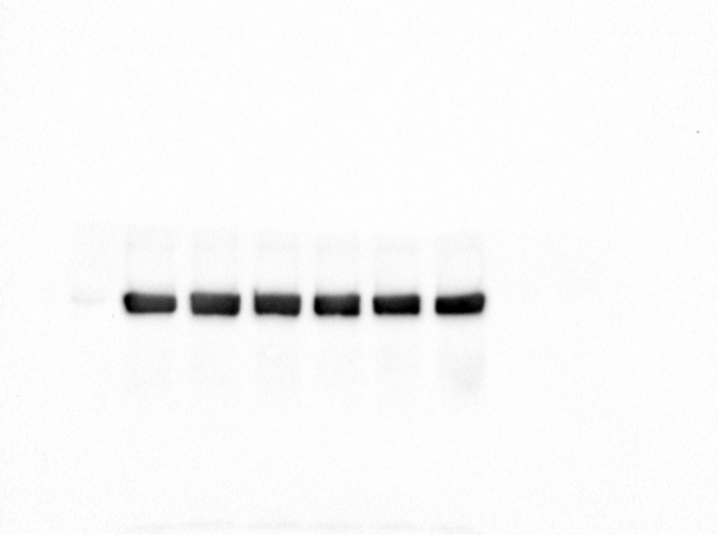

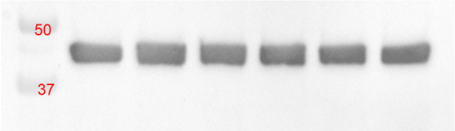

SUPPLEMENTARY REFERENCES

1. Cassidy PJ, Schneider JE, Grieve SM, Lygate C, Neubauer S, Clarke K. Assessment of motion gating strategies for mouse magnetic resonance at high magnetic fields. *J Magn Reson Imaging* 2004;**19**:229-237.
2. Gilchrist S, Kinchesh P, Kersemans V, Beech J, Allen D, Brady M, Vojnovic B, Schneider J, Miller J, Smart S. A simple, open and extensible gating Control unit for cardiac and respiratory synchronisation control in small animal MRI and demonstration of its robust performance in steady-state maintained CINE-MRI. *Magn Reson Imaging* 2021;**81**:1-9.
3. Lewis AJM, Miller JJ, Lau AZ, Curtis MK, Rider OJ, Choudhury RP, Neubauer S, Cunningham CH, Carr CA, Tyler DJ. Noninvasive Immunometabolic Cardiac Inflammation Imaging Using Hyperpolarized Magnetic Resonance. *Circ Res* 2018;**122**:1084-1093.
4. Miller JJ, Lau AZ, Teh I, Schneider JE, Kinchesh P, Smart S, Ball V, Sibson NR, Tyler DJ. Robust and high resolution hyperpolarized metabolic imaging of the rat heart at 7 T with 3D spectral-spatial EPI. *Magn Reson Med* 2016;**75**:1515-1524.
5. Miller JJ, Lau AZ, Tyler DJ. Susceptibility-induced distortion correction in hyperpolarized echo planar imaging. *Magn Reson Med* 2018;**79**:2135-2141.
6. Naressi A, Couturier C, Castang I, de Beer R, Graveron-Demilly D. Java-based graphical user interface for MRUI, a software package for quantitation of in vivo/medical magnetic resonance spectroscopy signals. *Comput Biol Med* 2001;**31**:269-286.
7. Schroeder MA, Swietach P, Atherton HJ, Gallagher FA, Lee P, Radda GK, Clarke K, Tyler DJ. Measuring intracellular pH in the heart using hyperpolarized carbon dioxide and bicarbonate: a 13C and 31P magnetic resonance spectroscopy study. *Cardiovasc Res* 2010;**86**:82-91.
8. van Hasselt JGC, Rahman R, Hansen J, Stern A, Shim JV, Xiong Y, Pickard A, Jayaraman G, Hu B, Mahajan M, Gallo JM, Goldfarb J, Sobie EA, Birtwistle MR, Schlessinger A, Azeloglu EU, Iyengar R. Transcriptomic profiling of human cardiac cells predicts protein kinase inhibitor-associated cardiotoxicity. *Nat Commun* 2020;**11**:4809.
